# Class III PI3K regulates organismal glucose homeostasis by providing negative feedback on hepatic insulin signalling

**DOI:** 10.1038/ncomms9283

**Published:** 2015-09-21

**Authors:** Ivan Nemazanyy, Guillaume Montagnac, Ryan C. Russell, Lucille Morzyglod, Anne-Françoise Burnol, Kun-Liang Guan, Mario Pende, Ganna Panasyuk

**Affiliations:** 1 Institut Necker-Enfants Malades (INEM), Cedex 14, 75993 Paris, France; 2 Institut National de la Santé et de la Recherche Médicale (INSERM), Cedex 14, U1151, 75993 Paris, France; 3 Université Paris Descartes, Sorbonne Paris Cité, 75006 Paris, France; 4 Institut National de la Santé et de la Recherche Médicale (INSERM), U1170, Gustave Roussy Institute, 94805 Villejuif, France; 5 Department of Pharmacology, University of California at San Diego, La Jolla, California 92093, USA; 6 Moores Cancer Center, University of California at San Diego, La Jolla, California 92093, USA; 7 Institut National de la Santé et de la Recherche Médicale (INSERM), U1016, Institut Cochin, 75014 Paris, France; 8 Centre national de la recherche scientifique (CNRS), UMR8104, 75014 Paris, France

## Abstract

Defective hepatic insulin receptor (IR) signalling is a pathogenic manifestation of metabolic disorders including obesity and diabetes. The endo/lysosomal trafficking system may coordinate insulin action and nutrient homeostasis by endocytosis of IR and the autophagic control of intracellular nutrient levels. Here we show that class III PI3K—a master regulator of endocytosis, endosomal sorting and autophagy—provides negative feedback on hepatic insulin signalling. The ultraviolet radiation resistance-associated gene protein (UVRAG)-associated class III PI3K complex interacts with IR and is stimulated by insulin treatment. Acute and chronic depletion of hepatic Vps15, the regulatory subunit of class III PI3K, increases insulin sensitivity and Akt signalling, an effect that requires functional IR. This is reflected by FoxO1-dependent transcriptional defects and blunted gluconeogenesis in *Vps15* mutant cells. On depletion of Vps15, the metabolic syndrome in genetic and diet-induced models of insulin resistance and diabetes is alleviated. Thus, feedback regulation of IR trafficking and function by class III PI3K may be a therapeutic target in metabolic conditions of insulin resistance.

## Abstract (web summary)

PI3K is activated as a result of insulin receptor (IR) signalling. Here the authors show that activation of specific class III PI3Ks in response to insulin promotes IR endocytosis and lysosomal degradation, providing negative feedback on IR signalling by reducing the time IR is activated.

## Graphical abstract

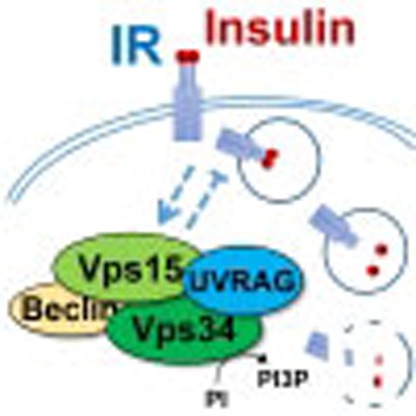

Catabolic and anabolic processes are tightly coordinated in response to nutrient and energy availability. The trafficking of endocytic and autophagic vacuoles to the lysosomal degradation system is a key event to satisfy the energetic needs of the cell after food intake or during starvation. Insulin, the major anabolic hormone in mammals, is released by pancreatic beta cells into the bloodstream after food intake and acts in peripheral tissues by binding transmembrane insulin receptor (IR) with tyrosine kinase activity^1^. The endocytosis of IR plays an important role in the magnitude and nature of insulin signals. Depending on the tissues, IR can be internalized at the caveolae, which are invaginations of the plasma membrane, or at clathrin-coated pits^2^,^3^,^4^. The internalization of IR and sorting to the lysosomes is a major pathway to degrade receptor and to remove hormone from the circulation thus terminating insulin action^1^,^5^. However, in the endosomes, IR maintains its activity and interacts with signal transduction elements^6^. Insulin receptor substrates (IRS1–4) act as signalling scaffolds at both the plasma membrane and endosomes, where essential proteins for the metabolic action of insulin are recruited, such as class I PI3K, IRS and Akt kinases^1^,^7^,^8^. In addition to being regulated by the endo/lysosomal system, insulin signalling tightly controls autophagy. Autophagy is a process by which proteins and organelles are engulfed in double-membrane autophagosomes and degraded after fusion with lysosomes^9^. Insulin inhibits the autophagic degradation of cellular components, while favouring nutrient uptake and usage from the extracellular milieu. Consistent with a central role in nutrient homeostasis, a growing body of evidence indicates that defects in the endocytosis and autophagy may contribute to metabolic syndromes^10^. Defects in IR internalization have been associated with obesity and type 2 diabetes (T2D), both in humans and in animal models^11^,^12^. Autophagy defects have a complex and controversial influence on insulin resistance and nutrient homeostasis^13^,^14^,^15^.

Among the molecular mechanisms coordinating endocytosis and autophagy, class III PI3K has a central evolutionarily conserved role. Class III PI3K is present in every eukaryotic cell, from yeast, in which it was initially discovered, to mammals^16^. It is constituted by a complex of the regulatory and catalytic subunits Vps15 and Vps34, respectively. Vps15 is a putative serine/threonine protein kinase, which is required for Vps34 stability, activity and membrane targeting^17^. The lipid kinase activity of Vps34 is a major source of phosphatidylinositol 3-phosphate (PI3P) in the cell, which functions as secondary messenger at the intracellular membranes and docking signal for proteins containing PI3P-binding domains, such as FYVE or PX (ref. 18). Binding to PI3P promotes the formation of protein scaffolds that are involved in multiple processes, including autophagy, trafficking from the plasma membrane towards the lysosome and endosomal sorting. Both autophagic and endocytic trafficking require class III PI3K activity. It is now becoming clear that class III PI3K contributes to these pleiotropic functions by engaging in distinct protein complexes. Binding of Atg14-related protein (ATG14) or ultraviolet radiation resistance-associated gene protein (UVRAG) to a Vps34/Vps15/Beclin-1 complex is mutually exclusive^19^,^20^. The ATG14-containing complex stimulates Vps34 activity at the phagophore membranes and is required for autophagy initiation in response to nutrient withdrawal. The UVRAG-containing complex is implicated in endosome and autophagosome maturation. The majority of Vps34/Vps15 in mammalian cells is not in complex with ATG14 and UVRAG, the existence of additional complexes is likely^21^.

Vps34/Vps15 activity is now thought to integrate environmental cues. Glucose starvation increases class III PI3K activity, in both ATG14- and UVRAG-containing complexes, through an AMP-activated kinase (AMPK)-dependent mechanism^22^. During conditions of amino-acid starvation that promote autophagy, class III PI3K activity in ATG14-containing complexes is specifically stimulated, whereas the majority of Vps34 complexes are inhibited. This is due to the ability of ATG14 to recruit the Ulk1 kinase, which is activated by amino-acid starvation and phosphorylates Beclin-1 to promote autophagy^23^. Less clear is the impact of insulin on class III PI3K activity. Although insulin does not appear to affect total class III PI3K activity^24^, the effect of insulin treatment on specific Vps34/Vps15 complexes has never been reported.

We show that UVRAG-containing class III PI3K complexes are effectors of insulin action that associate with IR. Moreover, perturbing class III PI3K activity has a major impact on the kinetics of IR degradation, as well as on downstream signal transduction. As a result, we demonstrate that targeting Vps15 has a beneficial metabolic effect in mouse models of obesity and T2D. These data define class III PI3K as a crucial element in the response to insulin and nutrients (glucose and amino acids) at the crossroad of autophagy and IR trafficking.

## Results

### Loss of *Vps15* delays IR degradation and promotes signalling

**Figure 1:**
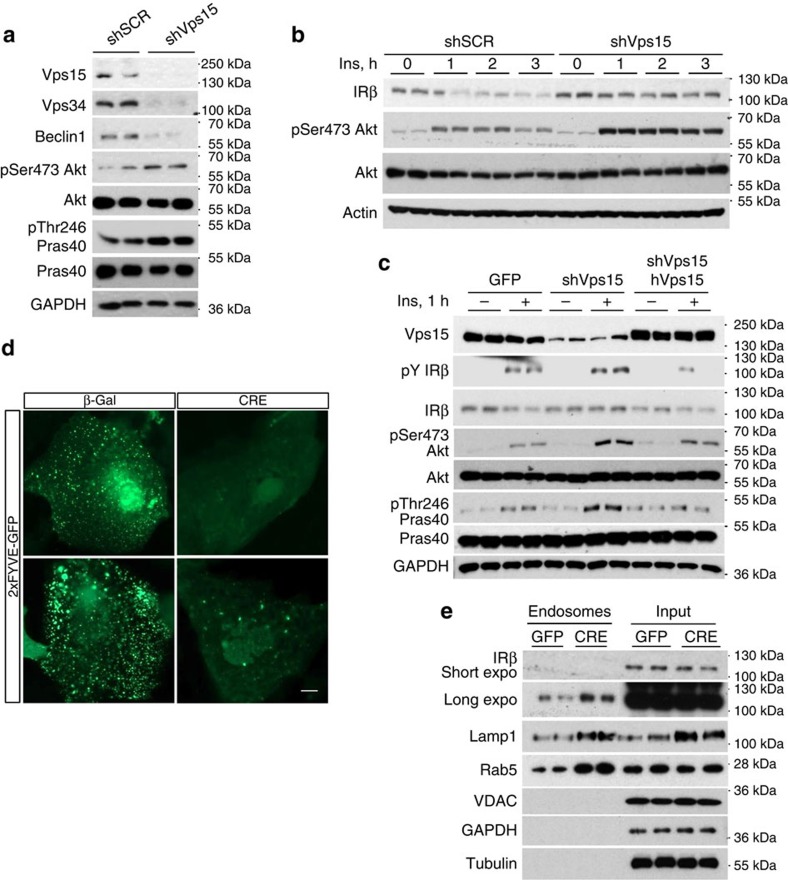
IR signalling is induced in Vps15-depleted cells. (**a**) Hepa1.6 cells were transduced with adenoviral vectors expressing shRNA Vps15 or shRNA SCR as a negative control. The 48-h post-transduction expression of components of class III PI3K and status of Akt-signalling pathway was revealed by immunoblot analysis. Immunoblot with anti-GAPDH antibodies was used as a loading control. Densitometric analyses of phosphorylated proteins normalized to total protein levels are presented on Supplementary Fig. 1a. (**b**) Hepa1.6 cells were transduced with shRNA Vps15- or shRNA SCR-expressing adenoviruses. The 24-h postinfection cells were serum starved for 24 h followed by stimulation with 1 μM insulin (Ins). IR levels and status of the pathway activation was determined by immunoblot analysis. Densitometric analyses of phosphorylated Akt normalized to total Akt level and actin-normalized IRβ levels are presented on Supplementary Fig. 1a. (**c**) Hepa1.6 cells were transduced with GFP, shRNA Vps15 or shRNA Vps15 in combination with hVps15 cDNA-expressing adenoviruses. The 24-h postinfection cells were serum starved for 24 h followed by stimulation with 1 μM insulin for 1 h. IR levels and status of Akt-signalling pathway activation was determined by immunoblot analysis. (**d**) Vps15^f/f^ primary hepatocytes were transduced with Adeno-β-Gal- or Adeno-CRE-expressing vectors. To detect endogenous PI3P, 48-h postinfection hepatocytes were transfected with the reporter plasmid expressing 2xFYVE-GFP fusion protein. Cells were kept in the nutrient-rich media supplemented with 10% FBS and insulin, 24-h post-transfection cells were PFA fixed and PI3P-positive compartments (endosomes) were visualized by confocal microscopy. Scale bar, 10 μm. (**e**) Vps15^f/f^ primary hepatocytes were transduced with Adeno-GFP or Adeno-CRE vectors. Cells were kept in the nutrient-rich media supplemented with 10% FBS and insulin. The 72-h post-transduction cells were collected and endosomal fraction prepared from the equal mass of cells. Equal volumes of endosomal fraction were loaded for Adeno-GFP or Adeno-CRE samples. Total protein extracts were prepared from the fraction of pelleted cells. The protein concentration in the extracts was measured by Bradford and the equal amount of proteins (10 μg) was loaded for each sample. Proteins present in endosomal fraction and in total extracts were revealed by immunoblot analysis. Immunoblot with anti-VDAC, anti-GAPDH and anti-Tubulin antibodies served as a control of purity of endosomal fraction for mitochondria proteins, cytoplasmic proteins and cytoskeletal proteins, respectively.

To analyse the role of class III PI3K in IR responses and metabolic homeostasis, we first depleted *Vps15* using a specific shRNA in mouse hepatocellular carcinoma Hepa1.6 cells. In line with its requirement for the class III PI3K complex stability^25^, the downregulation of Vps15 was paralleled by decreased levels of other core components of the complex—Vps34 and Beclin-1 (Fig. 1a). We revealed that Akt signalling, a major downstream effector of activated IR^8^, was upregulated in *Vps15*-depleted cells (Fig. 1a and Supplementary Fig. 1a), as indicated by increased phosphorylation of Akt and its substrate proline-rich Akt substrate of 40 kDa (Pras40) protein. These observations prompted us to hypothesize that IR turnover could be affected in *Vps15*-depleted cells. To test this possibility, we followed IR levels on insulin stimulation. In control scrambled hairpin (shSCR)-treated cells, 50% of the catalytic subunit of IR, IRβ, was degraded 3 h after insulin stimulation (Fig. 1b and Supplementary Fig. 1a), suggesting receptor internalization and degradation in the lysosomes on exposure to the agonist. Interestingly, depletion of *Vps15* impaired IRβ degradation, concomitant with increased insulin signalling, as measured by Akt phosphorylation (Fig. 1b and Supplementary Fig. 1a). Importantly, the effects of *Vps15* depletion on IR levels and downstream Akt activation could be rescued by overexpression of recombinant hVps15 protein resistant to shRNA (Fig. 1c). In addition, the improved insulin signalling in *Vps15*-depleted cells was evidenced by increased complexes between IR and its downstream effectors (Supplementary Fig. 1b,c). Next, we analysed primary hepatocytes from Vps15^f/f^ mice transduced with Cre recombinase-expressing adenoviral vector. As previously observed^26^, CRE-mediated recombination of exon 2 in *Vps15* gene led to the expression of non-functional truncated Vps15 protein due to the usage of alternative start codon in the exon 4 (Supplementary Fig. 2a). As expected, depletion of *Vps15* in hepatocytes resulted in loss of class III PI3K complex expression (Supplementary Fig. 2a). Importantly, IR levels decreased in control hepatocytes on insulin stimulation, while they remained constant in mutant cells (Supplementary Fig. 2b,c). Importantly, the insulin-stimulated IR degradation to large extend could be rescued by inhibition of lysosomal activity confirming that the lysosomal pathway is a major route of receptor degradation (Supplementary Fig. 2b). The lower basal levels of IRβ in primary cultures of *Vps15*-depleted hepatocytes likely reflected compensatory mechanisms due to persistent pathway activation. Consistent with the results in Hepa1.6 cells, insulin-stimulated Akt phosphorylation was also upregulated in *Vps15*-depleted hepatocytes (Supplementary Fig. 2c). In sum, both in hepatocellular carcinoma cells and in primary hepatocytes *Vps15* depletion interferes with IR degradation and results in improved Akt signalling.

### *Vps15* inactivation results in accumulation of the endosomes

Class III PI3K-dependent production of PI3P on endosomes is required for the recruitment of PI3P-binding domain-containing proteins that regulate different steps of endocytosis. In agreement, *Vps15* depletion impaired the recruitment of the PI3P probe 2xFYVE to the endosomal compartment, providing additional evidence for the loss of class III PI3K activity in *Vps15*-depleted cells (Fig. 1d). Perturbed endosomal plasticity on *Vps15* loss in primary hepatocytes was further evidenced by striking vacuolization and expansion of the Lamp1-positive compartment (Supplementary Fig. 2d). Similarly, the immunofluorescent studies of Hepa1.6 cells revealed profound perturbations of endolysosomal compartment on short-term Vps15 downregulation (Supplementary Fig. 1d). In addition, endosome purification by differential centrifugation in sucrose gradient revealed increased levels of endosomal markers in *Vps15* mutant hepatocytes, as assessed by the immunoblot analysis of the early and late endosomal proteins Rab5 and Lamp1, respectively (Fig. 1e). Of note, in *Vps15*-depleted cells compared with green fluorescent protein (GFP)-transduced cells, IRβ levels were enriched in the endosomal fraction unlike in total cell extracts.

### Slow kinetics of IR trafficking in Vps15-depleted cells

**Figure 2:**
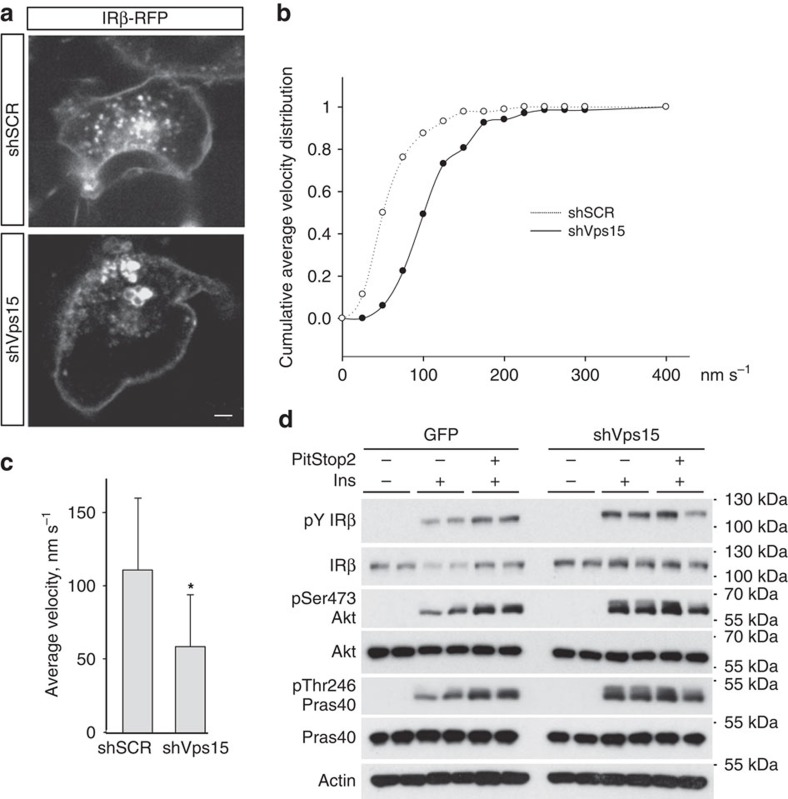
IR trafficking is impaired on class III PI3K inactivation. (**a**) Hepa1.6 cells were transduced with shRNA Vps15- or shRNA SCR-expressing adenoviruses. IRβ-RFP was overexpressed 24 h postinfection and cells were imaged 24 h post-transfection. Representative spinning disc confocal snap shot image of the live cell used for endosome tracking is presented (the corresponding movie is available as a Supplementary Information File). Endosomes were manually tracked using MetaMorph software. Cumulative endosome velocity (**b**) and average endosome velocity (**c**) were calculated for each endosome. Data are means±s.d. (*n*=95–125 endosomes, *P<0.001 versus Adeno-shRNA SCR, Mann–Whitney Rank-sum test). (**d**) Hepa1.6 cells were transduced with shRNA Vps15- or GFP-expressing adenoviruses. The 24-h postinfection cells were serum starved for 24 h followed by stimulation with 1 μM insulin for 2 h. Before stimulation with insulin, cells were pretreated with 3 μM of PitStop2 inhibitor for 15 min. IRβ levels and status of the pathway activation was determined by immunoblot analysis. Densitometric analyses of phosphorylated Akt and Pras40 normalized to levels of total proteins are presented on Supplementary Fig. 1e.

To study the dynamics of IR trafficking we transiently overexpressed red fluorescent protein (RFP)-tagged human IRβ. RFP-IRβ was detected at the plasma membrane and in small endosomes in control and *Vps15*-depleted Hepa1.6 cells (Fig. 2a). RFP-IRβ was also present on enlarged vacuolar endosomes uniquely in *Vps15*-depleted cells, suggesting strong endocytic trafficking defects of the receptor (Fig. 2a). We performed live cell imaging of these cells to track RFP-IRβ-labelled endosomes and quantified their movement parameters (Supplementary Movie). The tracking of RFP-IRβ-positive small endosomes revealed that *Vps15* inactivation resulted in a global reduction of the velocity of this population (Fig. 2b). This was further reflected by a 50% decrease in the average velocity of RFP-IRβ-positive endosomes (Fig. 2c). Together, these data reveal the accumulation of IR on enlarged and static endosomes on *Vps15* depletion. These observations also suggest that defective IR degradation observed in *Vps15*-inactivated cells is a direct consequence of trafficking defect that delays receptor delivery into lysosomes.

### Block of endocytosis improves IR signalling

To confirm that defects in receptor degradation lead to improved IR signalling in *Vps15*-depleted cells, IR internalization and trafficking was blocked using the selective inhibitor of clathrin function, PitStop2 (ref. 27). As expected, endocytosis inhibition in control cells largely prevented IRβ degradation resulting in increased IRβ tyrosine phosphorylation (Fig. 2d). On the contrary, clathrin inhibition had no effect on IRβ stability and phosphorylation in *Vps15*-depleted cells. Importantly, the interference with IRβ endocytic trafficking and degradation in control cells mimicked the effect of *Vps15* depletion on insulin signalling as evidenced by increased Akt phosphorylation and phosphorylation of its downstream target Pras40 (Fig. 2d and Supplementary Fig. 1e). Altogether, these results demonstrate that IR signalling is potentiated on class III PI3K inactivation in concomitance with altered kinetics of IR endocytic trafficking.

### Insulin stimulates UVRAG-associated class III PI3K activity

**Figure 3:**
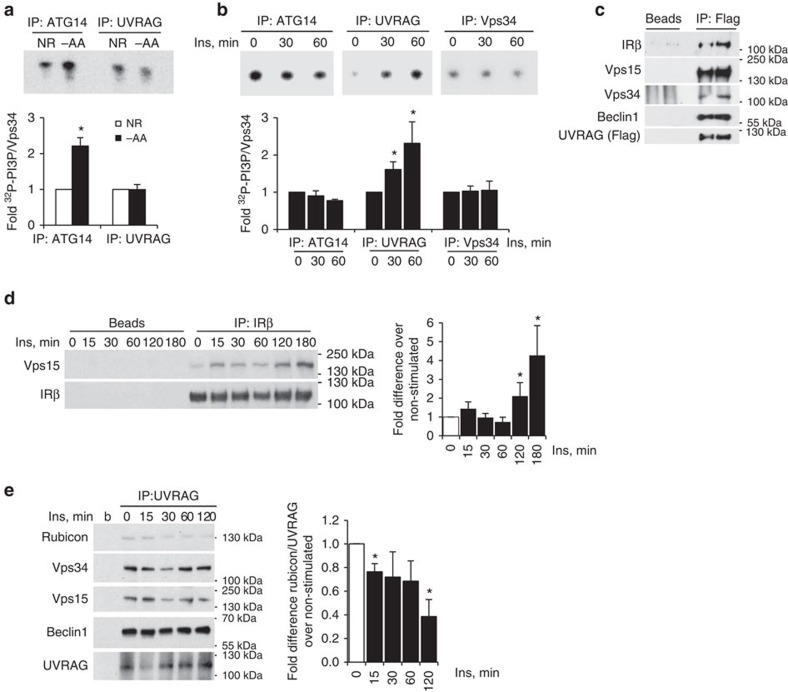
UVRAG-associated class III lipid kinase is activated by insulin. (**a**) ATG14- and UVRAG-containing Vps34 complexes were immunoprecipitated from Hepa1.6 cells grown in the presence (NR) or absence of amino acids (−AA) and assayed for lipid kinase activity. Inputs for each assay were immunoblotted to determine the amounts of the Vps34 co-immunoprecipitated. PI3P signals were densitometrically measured and normalized to Vps34 protein levels. The data are presented as a fold difference of PI3P normalized to co-immunoprecipitated Vps34 levels revealed by immunoblot for each condition. Data are means±s.e.m. (*n*=4, *P<0.05 versus NR, two-tailed, unpaired Student’s *t*-test). (**b**) ATG14- and UVRAG-containing Vps34 complexes were immunoprecipitated from primary hepatocytes, which were serum starved for 24 h followed by stimulation with 1 μM insulin (Ins) and assayed for lipid kinase activity. PI3P signals were densitometrically measured. The data are presented as a fold difference of PI3P normalized to co-immunoprecipitated Vps34 levels revealed by immunoblot for each condition. Data are means±s.e.m. (*n*=3, *P<0.05 versus starved cells, two-tailed, unpaired Student’s *t*-test). Representative immunoblot analyses of ATG14, UVRAG and Vps34 co-immunoprecipitates are presented as Supplementary Fig. 3a and Supplementary Fig. 3b. (**c**) Primary hepatocytes were transiently transfected with UVRAG–Flag cDNA-expressing vector 12 h post-plating and were kept in the serum containing media. The 36-h post-transfection cells were collected and UVRAG–Flag complexes were immunoprecipitated with anti-Flag antibody. The presence of IRβ and class III PI3K subunits in the immunoprecipitation eluates was revealed by immunoblot. (**d**) Endogenous IRβ was immunoprecipitated from primary hepatocytes, which were serum starved for 24 h and stimulated with 1 μM insulin. The presence of Vps15 and IRβ in the immunoprecipitation eluates was revealed by immunoblot. Densitometric analyses of co-immunoprecipitated endogenous Vps15 normalized to IRβ are presented as fold difference over the unstimulated condition. Data are means±s.e.m. (*n*=3, *P<0.05 versus starved cells, two-tailed, unpaired Student’s *t*-test). (**e**) UVRAG-containing complexes were immunoprecipitated from primary hepatocytes, which were serum starved for 24 h followed by stimulation with 1 μM insulin for indicated times. Immunoprecipitated endogenous class III PI3K subunits were revealed by immunoblotting. Densitometric analyses of co-immunoprecipitated endogenous Rubicon normalized to UVRAG are presented as fold difference over the unstimulated condition. Data are means±s.e.m. (*n*=3, *P<0.05 versus starved cells, two-tailed, unpaired Student’s *t*-test). The protein G beads served as a control of the nonspecific binding in **c**–**e**.

Since IR signalling is negatively regulated by class III PI3K, we asked whether the activity of class III PI3K could be sensitive to insulin. While an increase in cellular PI3P levels has been reported, no significant changes in hVps34 activity on insulin stimulation have been observed^24^,^28^,^29^. Recent reports demonstrated that measurement of total class III PI3K activity immunoprecipitated with anti-Vps34 antibody is not informative as Vps15/Vps34 proteins are engaged in complexes with different functions and regulation^21^. We measured class III PI3K activity associated with ATG14 and UVRAG, two mutually exclusive binding proteins of the core Vps34/Vps15 complex (Supplementary Fig. 3a). In agreement with recent reports^22^ and consistent with autophagy activation, Vps34 activity in ATG14 complex was significantly increased in response to amino-acid withdrawal (Fig. 3a). At the same time, UVRAG-associated Vps34 activity was insensitive to amino-acid deprivation (Fig. 3a). To determine whether class III PI3K activity is regulated by insulin, we analysed total Vps34 and UVRAG- or ATG14-associated Vps34 lipid kinase activity in primary hepatocytes, which were insulin starved and then stimulated for different times. Consistent with earlier reports, total Vps34 activity was not modified in response to insulin (Fig. 3b). Measurements of ATG14-associated Vps34 lipid kinase activity revealed that in contrast to amino-acid starvation, it was unchanged by insulin stimulation. However, insulin stimulation resulted in twofold increase of Vps34 activity in UVRAG-containing complexes (Fig. 3b). In addition, co-immunoprecipitation studies revealed that IR interacts with ectopically expressed Flag-tagged UVRAG complexes (Fig. 3c). Further studies revealed that in primary hepatocytes complex between endogenous IR and regulatory subunits of class III PI3K, Vps15 and UVRAG was induced by insulin stimulation (Fig. 3d and Supplementary Fig. 3c). Immunoprecipitation experiments followed by class III PI3K biochemical assay further confirmed that active Vps34 was in complex with endogenous IR (Supplementary Fig. 3d). Mechanistically, insulin stimulation of primary hepatocytes resulted in dissociation of Rubicon, a negative regulator of Vps34 activity in UVRAG-containing complexes (Fig. 3e). Altogether, these data demonstrate that distinct class III PI3K complexes are differently regulated in response to nutrient starvation and insulin stimulation revealing unappreciated crosstalk between IR signalling and class III PI3K activity regulation.

### Hepatic Vps15 depletion improves glucose metabolism

**Figure 4:**
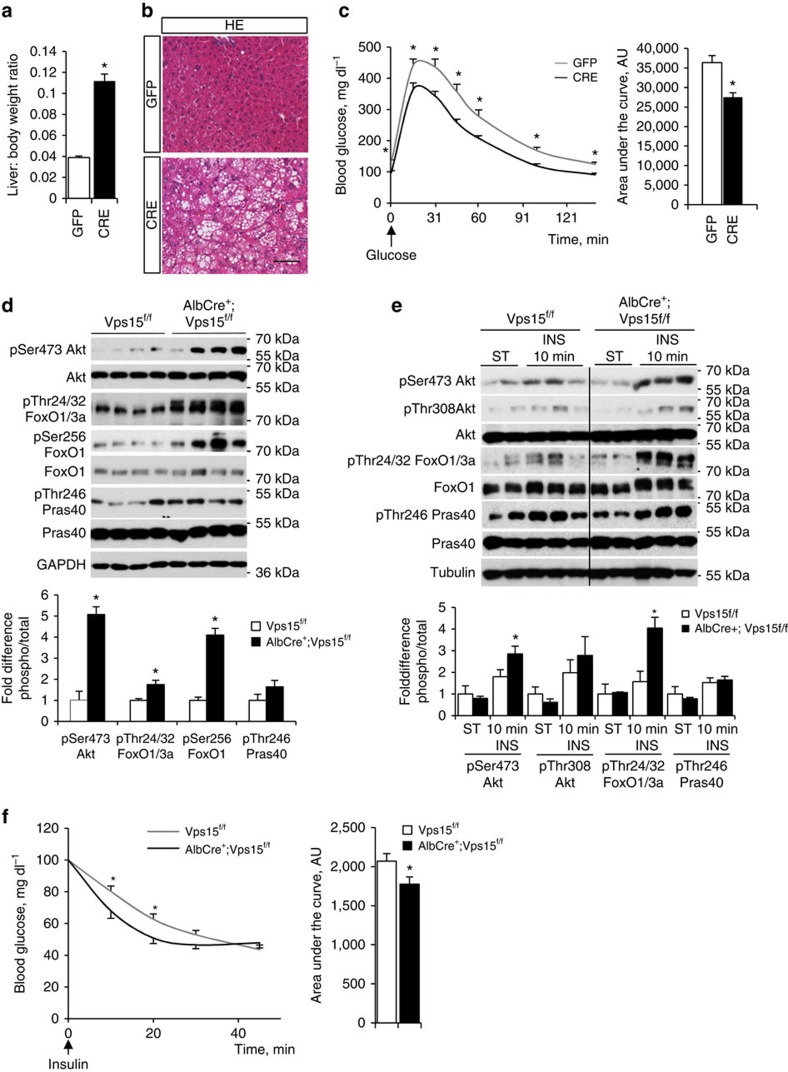
Increased insulin action in *Vps15* hepatic mutants. (**a**) Liver weight to body weight ratio of random-fed Vps15^f/f^ mice 10 days post-transduction with Adeno-GFP or Adeno-CRE vectors. Data are means±s.e.m. (*n*=5–6, *P<0.05: versus Adeno-GFP, two-tailed, unpaired Student’s *t*-test). (**b**) H&E-stained liver sections of random-fed Vps15^f/f^ mice 10 days post-transduction with Adeno-GFP or Adeno-CRE vectors showing marked vacuolization of hepatocytes in livers of Adeno-CRE-transduced mice. Scale bar, 100 μm. (**c**) Intraperitoneal GTT in overnight fasted Vps15^f/f^ mice 7 days post-transduction with Adeno-GFP or Adeno-CRE vectors. The histogram of the average value of the area under the respective curve calculated with GraphPadPrizm5 software is presented. Data are means±s.e.m. (*n*=5–6, *P<0.05 versus Adeno-GFP, two-tailed, unpaired Student’s *t*-test). (**d**) Immunoblot analysis of total protein liver extracts of random-fed 2-month-old Vps15^f/f^ and AlbCre^+^;Vps15^f/f^ using indicated antibodies. Densitometric analyses of phosphoprotein levels normalized to total protein presented as folds over Vps15^f/f^. Data are means±s.e.m. (*n*=4–5, *P<0.05 versus Vps15^f/f^, two-tailed, unpaired Student’s *t*-test). (**e**) Immunoblot analysis of total protein liver extracts of starved for 12 h or starved for 12 h followed by injection of insulin 2-month-old Vps15^f/f^ and AlbCre^+^;Vps15^f/f^ mice. Densitometric analyses of phosphoprotein levels normalized to total protein presented as folds over starved Vps15^f/f^. Data are means±s.e.m. (*n*=3, *P<0.05: versus Vps15^f/f^, two-tailed, unpaired Student’s *t*-test). (**f**) Intraperitoneal insulin tolerance test performed on starved for 6 h 6-week-old Vps15^f/f^ and AlbCre^+^;Vps15^f/f^ mice. The histogram of an average value of the area under the respective curve was calculated with GraphPadPrizm5 software. Data are means±s.e.m. (*n*=8–14, *P<0.05 versus Vps15^f/f^, two-tailed, unpaired Student’s *t*-test).

Next, we sought to determine the consequences of class III PI3K inactivation on IR signalling and metabolic homeostasis *in vivo*. We recently demonstrated that *Vps15* is an essential gene product, as witnessed by the early embryonic lethality of whole body *Vps15* knockout mutants^26^. To circumvent the embryonic lethality and to address the role of Vps15 in tissue insulin response, hepatic *Vps15* was targeted *in vivo* in adult mice by CRE recombinase delivered through intravenous injection of adenoviral vectors. The selectiveness of this approach to hepatic tissue was evidenced by lack of *Vps15* gene recombination and unaffected Vps15 protein expression in other tissues (Supplementary Fig. 4a,b). Effective depletion of hepatic *Vps15* expression was confirmed both at transcript and protein levels revealing an 80% decrease of Vps15 levels in CRE-expressing livers (Supplementary Fig. 4c–e). Analysis of Vps15^f/f^ mice 10 days postinjection revealed that adenoviral CRE-treated mice developed liver hypertrophy (Fig. 4a and Supplementary Fig. 4f). Liver hypertrophy of *Vps15*-depleted mice was due to increase in hepatocyte cell size and cell number (Fig. 4b and Supplementary Fig. 4g). In addition, hepatocytes in adenoviral CRE-transduced livers developed striking vacuolization (Fig. 4b). In agreement with the requirement of class III PI3K for autophagy, the acute deletion of Vps15 resulted in autophagy block, as revealed by the accumulation of an autophagy cargo receptor, p62/SQSTM1, increased levels of the unconjugated form of LC3 (LC3-I) and a reduction of lipidated LC3 (LC3-II) (Supplementary Fig. 4c). Consistent with the data in cultured cells, hepatic Akt signalling was activated in *Vps15* mutant mice under refeeding conditions (Supplementary Fig. 5a). Importantly, acute short-term hepatic depletion of *Vps15* affected whole-body glucose metabolism, as adenoviral CRE-injected mice showed significant hypoglycaemia in starvation conditions and in response to an intraperitoneal glucose challenge (Fig. 4c). In sum, similarly to *in vitro* models, short-term depletion of *Vps15* *in vivo* potentiates insulin signalling and impacts whole-body glucose metabolism.

### Akt signalling is augmented in hepatic mutants of Vps15

Next, we generated the liver-specific *Vps15* knockout mice (herein referred as Vps15 LKO). To obtain Vps15 LKO mice, the Vps15^f/f^ mice were crossed with transgenic line overexpressing CRE under the mouse albumin enhancer/promoter. Alb-Cre drives liver-specific CRE expression starting at E13.5 and achieves efficient deletion of targeted gene at early postnatal stage both in hepatocytes and biliary cells^30^. The loss of hepatic *Vps15* expression was evidenced by transcript and protein analysis of the livers of Vps15 LKO mice (Supplementary Fig. 6a,b). Vps15 LKO mice recapitulated the main features observed after short-term *Vps15* depletion by adenoviral CRE transduction: hepatomegaly due to increased cell size and cell proliferation (Supplementary Fig. 6c–e), vacuolization (Supplementary Fig. 6f) and autophagy block witnessed by accumulation of p62, LC3 (LC3-I/II) and polyubiquitinated proteins (Supplementary Fig. 6b). Consistent with the observations in Hepa1.6 cells (Fig. 2a–d and Supplementary Fig. 1d), the IR trafficking defects in mutant hepatocytes were highlighted by the perturbations of endolysosomal compartment and accumulation of endogenous IR in *Vps15*-null hepatocytes (Supplementary Fig. 6g). Altogether, Vps15 LKO recapitulates the autophagy and IR trafficking defects observed both *in vitro* and *in vivo* on *Vps15* depletion.

Importantly, increased hepatic insulin signalling was observed in Vps15 LKO mice both in random-fed and on insulin challenge conditions, as witnessed by Akt and FoxO1 phosphorylation (Fig. 4d,e). Similarly to cell cultures, increased IR tyrosine phosphorylation was detected in the liver extracts of *Vps15* mutants compared with controls (Supplementary Fig. 7a). These data were consistent with the improved insulin and glucose tolerance after an intraperitoneal load observed in hepatic Vps15 LKO mice (Fig. 4f and Supplementary Fig. 7b). Interestingly, despite the autophagy block the hepatic *Vps15* mutant did not present with steatosis as reported for other mouse models of deficient autophagy^14^,^31^,^32^. On the contrary, the hepatic tissue of mice after chronic (Supplementary Fig. 8a,b) or acute (Supplementary Fig. 8e,f) *Vps15* depletion was characterized by significantly lower hepatic lipid content. This was accompanied by decreased expression of lipogenic enzymes and marked increase in expression of lipases (Supplementary Fig. 8c). In addition, loss of hepatic *Vps15* resulted in potent induction of glycolytic enzyme expression both on transcript and protein levels (Supplementary Fig. 9). The switch in expression of hepatic isoforms of hexokinase and pyruvate kinase to HK2 and PKM2 was observed in the livers of *Vps15* mutants (Supplementary Fig. 9). Given increased hepatic insulin signalling and striking rearrangements in the expression of metabolic enzymes observed on hepatic *Vps15* depletion, we compared the metabolic rates of age matched controls and Vps15 LKO mice. Vps15 LKO mice compared with littermate controls showed consistently higher oxygen consumption and energy expenditure in course of measurement (Supplementary Fig. 10a,b). This was accompanied by significantly augmented respiratory exchange ratio in *Vps15* knockouts compared with controls suggesting the preferable utilization of carbohydrates as an energy source (Supplementary Fig. 10c). In addition, we did not observe significant difference in cumulative food or drink intake between experimental groups (Supplementary Fig. 10d). The increased metabolic activity of hepatic *Vps15* mutants was most evident during the light phase of the cycle, which is in agreement with the increased activity of *Vps15* mutant mice observed (Supplementary Fig. 10e).

The ameliorated metabolic parameters of Vps15 LKOs were further observed under nutrient challenge, where *Vps15* control and mutant mice were submitted to 2-week long high-fat diet (HFD) regimen. As expected, after 2 weeks of HFD, the control mice developed significant glucose intolerance (Supplementary Fig. 7b). Remarkably, hepatic *Vps15* mutants, unlike controls, were protected from deleterious metabolic effect of HFD (Supplementary Fig. 7b). Improved insulin sensitivity on hepatic *Vps15* loss was also accompanied by significantly lower circulating levels of insulin both on chow and HFD in random-fed Vps15 LKO mice (Supplementary Fig. 7c).

Earlier studies addressed the causative link between the impaired autophagic flux and insulin resistance^14^. The study by Yang *et al*.^14^ demonstrated that the metabolic phenotype of *ob/ob* mice could be improved by increasing autophagic flux through ATG7 overexpression. In a somewhat contradictory fashion, recent work by Kim *et al*. suggested that the block of autophagy flux in *Atg7* skeletal muscle or liver mutants led to endoplasmic reticulum (ER) stress and resulted in activation of ATF4 transcription factor responses, leading to the induction of Fgf21 and protection of mice from the detrimental effects of HFD feeding^13^. In Vps15 LKO mice, we did not observe induction of hepatic Fgf21 mRNA expression (Supplementary Fig. 7d). In addition, Fgf21 levels measured in plasma of control and Vps15 LKO mice did not reveal significant differences between two genotypes in random-fed or in starved conditions (Supplementary Fig. 7e).

### Defective gluconeogenesis in *Vps15* mutants

**Figure 5:**
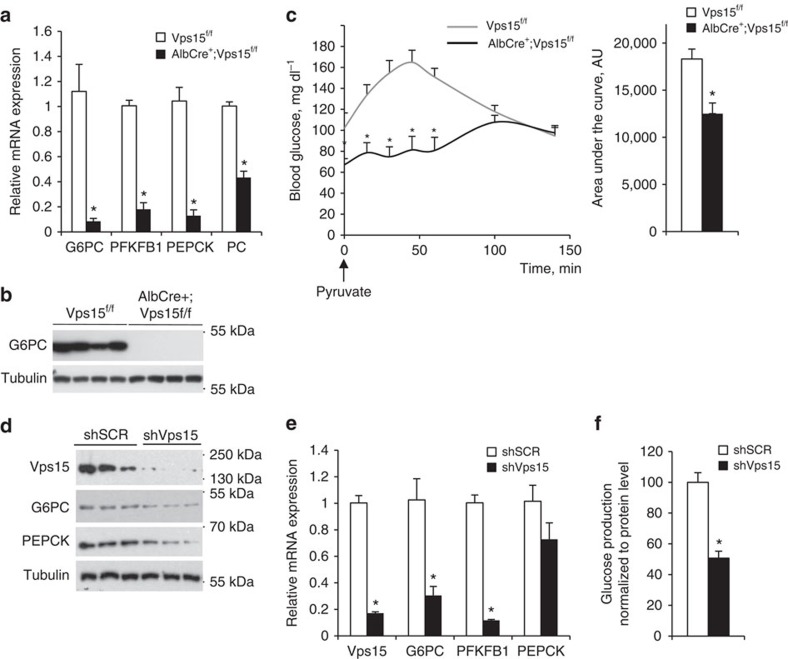
Gluconeogenesis is impaired in hepatic *Vps15* mutants. (**a**) Relative mRNA expression levels of genes implicated in gluconeogenesis in the livers of 1-month-old random-fed Vps15^f/f^ and AlbCre^+^;Vps15^f/f^ mice. Data are means±s.e.m. (*n*=5–8, *P<0.05 versus Vps15^f/f^, two-tailed, unpaired Student’s *t*-test). (**b**) Immunoblot analysis of total protein liver extracts of random-fed 1-month-old Vps15^f/f^ and AlbCre^+^;Vps15^f/f^ using G6PC antibody. Immunoblot with anti-tubulin antibody served as loading control. (**c**) Intraperitoneal pyruvate tolerance test performed on overnight-starved 6-week-old Vps15^f/f^ and AlbCre^+^;Vps15^f/f^ mice (*n*=5–8, *P<0.05 versus Vps15^f/f^, two-tailed, unpaired Student’s *t*-test). The histogram of an average value of the area under the respective curve was calculated with GraphPadPrizm5 software (*n*=5–8, *P<0.05 versus Vps15^f/f^, two-tailed, unpaired Student’s *t*-test). (**d**) Immunoblot analyses of cell extracts of primary hepatocytes transduced with Adeno-shRNA SCR or Adeno-shRNA Vps15 vectors with indicated antibodies. Primary hepatocytes were transduced and cells were collected 48 h postinfection for further analysis. Immunoblot with anti-tubulin antibody served as a loading control. (**e**) Relative mRNA expression levels of gluconeogenic enzymes in primary hepatocytes transduced with Adeno-shRNA SCR or Adeno-shRNA Vps15 vectors. Cells were collected 48 h postinfection. Data are means±s.e.m. (*n*=3, *P<0.05 versus shRNA SCR infected, two-tailed, unpaired Student’s *t*-test). (**f**) Glucose production by primary hepatocytes transduced with Adeno-shRNA SCR or Adeno-shRNA Vps15 vectors. Primary hepatocytes 48 h postinfection were incubated for 12 h in glucose-free DMEM containing lactate/pyruvate (10:1 mM). Glucose content in the media was assayed enzymatically and normalized to protein content. Data are means±s.e.m. (*n*=6, *P<0.05 versus shRNA SCR infected, two-tailed, unpaired Student’s *t*-test).

The suppression of gluconeogenesis is a major hypoglycaemic action of insulin in liver. To elucidate the mechanisms of improved glucose tolerance in Vps15 LKO, the expression of gluconeogenic enzymes was determined. Expression of key gluconeogenic enzymes was severely suppressed in both models of *Vps15* deficiency, in Vps15 LKO mice (Fig. 5a,b) and after short-term depletion by adenoviral CRE transduction (Supplementary Fig. 11a,b). Furthermore, the expression of peroxisome proliferator-activated receptor gamma coactivator 1 alpha (PGC1α), a transcriptional coactivator that is essential for the expression of genes of the gluconeogenesis pathway, was significantly downregulated in the livers of floxed mice injected with Adeno-CRE vectors (Supplementary Fig. 5b). In addition, hepatic expression of PGC1α as well as the gluconeogenic enzymes was inhibited even in starved mice and to great extent resistant to refeeding after *Vps15* depletion (Supplementary Fig. 5b). Functionally, pyruvate tolerance test in Vps15 LKO mice revealed a profound defect in glucose production in response to a pyruvate challenge (Fig. 5c). Next, to confirm the effects of class III PI3K inactivation on glucose metabolism in a cell autonomous model, *Vps15* was depleted in primary hepatocytes using adenoviral vectors expressing shRNA. As shown in Fig. 5d,e, *Vps15* knockdown in primary wild-type hepatocytes significantly reduced expression of rate-limiting enzymes in the gluconeogenesis pathway, including G6PC and PEPCK. The later effect could be rescued by concomitant overexpression of shRNA-resistant hVps15 (Supplementary Fig. 11c). Notably, the acute depletion of *Vps15* in primary hepatocytes was sufficient to inhibit gluconeogenesis, as evidenced by the drop in glucose release in response to pyruvate/lactate addition (Fig. 5f). Thus, interference with *Vps15* expression *in vitro* and *in vivo* is associated with impaired gluconeogenesis.

**Figure 6:**
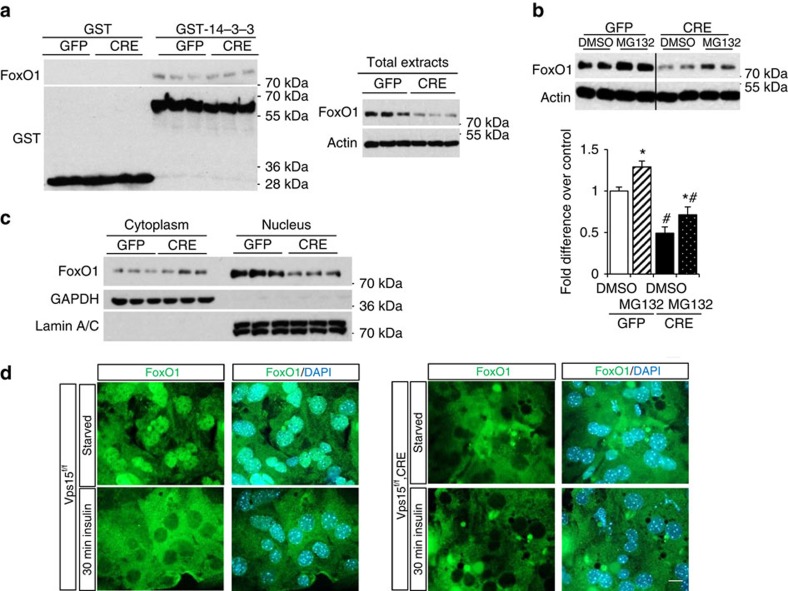
FoxO1 expression and action is compromised in *Vps15*-depleted hepatocytes. (**a**) Immunoblot analyses of GST-Sepharose eluates with anti-FoxO1 antibody (left panel). Recombinant 14-3-3-GST or GST proteins (2 μg) were coupled to Glutathione Sepharose 4B beads followed by incubation with 800 μg of total protein extracts of control or *Vps15*-depleted primary hepatocytes, which were collected 72 h post-transduction with Adeno-GFP or Adeno-CRE vectors. Decreased expression levels of FoxO1 protein was detected in total extracts of Adeno-CRE-infected hepatocytes used for the pull-down assay (right panel). (**b**) FoxO1 protein levels could be partially rescued by MG132 treatment of *Vps15*-depleted hepatocytes. After 72 h of transduction with Adeno-GFP or Adeno-CRE vectors hepatocytes were treated with 10 μM MG132 for 1 h before collected for further analysis. Immunoblot analysis of FoxO1 protein levels is presented. Immunoblot with anti-actin antibody serves as a loading control. Densitometric analyses of actin-normalized FoxO1 protein levels presented as folds over Adeno-GFP-transduced hepatocytes. Data are means±s.e.m. (*n*=3, *P<0.05 versus dimethylsulfoxide (DMSO), ^#^*P*<0.05 versus GFP, two-tailed, unpaired Student’s *t*-test). (**c**) Immunoblot analyses of nuclear and cytoplasmic fractions of control and *Vps15*-depleted primary hepatocytes 72 h post-transduction with Adeno-GFP or Adeno-CRE vectors with indicated antibodies. GAPDH and Lamin A/C are used as controls for cross-contamination of cytoplasmic and nuclear fractions, respectively. (**d**) Immunofluorescent analyses of FoxO1 subcellular localization in control and *Vps15*-depleted primary hepatocytes 72 h post-transduction with Adeno-GFP or Adeno-CRE vectors. Before fixation cells were either stimulated with1 μM insulin for 30 min or kept untreated in media without serum. Cells were PFA fixed and stained with anti-FoxO1 antibody, secondary anti-rabbit IgG Alexa Fluor 565 antibody was used for detection (images presented in green pseudocolour). Scale bar, 20 μm.

The striking defect in gluconeogenic gene expression in *Vps15* mutants phenocopies the changes in the hepatic metabolism observed in FoxO1 LKO mice^33^,^34^. FoxO1 is a master regulator of carbohydrate metabolism by positively controlling the expression of key gluconeogenesis enzymes G6PC and PEPCK^34^,^35^. Consistently, transcriptional activity of FoxO1 is under negative control of insulin receptor signalling via Akt-mediated phosphorylation^36^. FoxO1 phosphorylation by Akt promotes its interaction with 14-3-3 proteins, nuclear exclusion and cytosolic degradation by the proteasome^37^,^38^. In line with the increased stability of IR, Akt activation and increased FoxO1 phosphorylation observed in hepatic *Vps15* mutants, FoxO1 levels were markedly decreased, while the interaction with 14-3-3 proteins was maintained in *Vps15*-depleted hepatocytes (Fig. 6a). Furthermore, FoxO1 protein levels in *Vps15*-depleted hepatocytes were partly rescued by treatment with the proteasome inhibitor MG132, suggesting an active proteasomal degradation of FoxO1 protein in mutant hepatocytes (Fig. 6b). Notably, a predominant cytoplasmic localization of FoxO1 was observed in *Vps15* mutant hepatocytes by fractionation and immunofluorescence experiments (Fig. 6c,d). These results demonstrate that class III PI3K activity has a cell autonomous role in insulin signal transduction, FoxO1-dependent transcription and gluconeogenic programme.

### Improved Akt signalling on *Vps15* depletion requires IR

**Figure 7:**
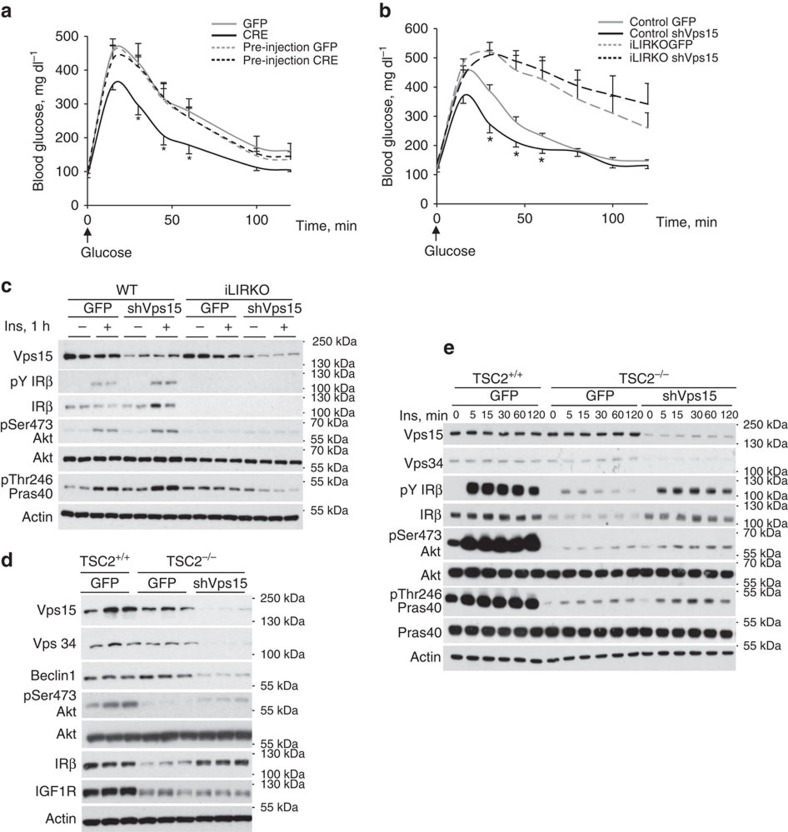
Akt activation in cells depleted of Vps15 requires functional IR. (**a**) Vps15^f/f^ mice were submitted to HFD regime during 2 weeks followed by intraperitoneal GTT after overnight fast (pre-injection GTT or pre-injection CRE). Next, mice were divided into two groups and were transduced with either Adeno-GFP- or Adeno-CRE-expressing vectors. Five days post-transduction GTT was performed after overnight fast. Data are means±s.e.m. (*n*=5–6, *P<0.05 versus Adeno-GFP, two-tailed, unpaired Student’s *t*-test). (**b**) Three-month-old iLIRKO and control mice were injected with tamoxifen to induce the IR deletion. Two-month postinjection iLIRKO and control mice were transduced with Adeno-GFP or Adeno-shRNA Vps15 vectors. Intraperitoneal GTT was performed in overnight-starved control and iLIRKO mice 5 days post-transduction. Mice were injected with 2 g kg^−1^ of glucose and glycaemia measured at indicated times. Data are means±s.e.m. (*n*=6–9, *P<0.05 versus Adeno-GFP, two-tailed, unpaired Student’s *t*-test). (**c**) Primary hepatocytes prepared from control or iLIRKO mutant mice were transduced with Adeno-GFP or Adeno-shRNA Vps15 vectors. The 24-h post-transduction cells were starved and then stimulated with 1 μM insulin (Ins) for 1 h. Activation of IR-signalling pathway was revealed by immunoblot analysis. (**d**) Immortalized TSC2^−/−^;p53^−/−^ and control TSC2^+/+^;p53^−/−^ MEFs were transduced with Adeno-GFP or Adeno-shRNA Vps15 vectors. Cells were kept in the media in presence of 10% serum and were collected for analysis 24 h postinfection. Total protein extracts were analysed by immunoblotting. (**e**) Immortalized TSC2^−/−^;p53^−/−^ and control TSC2^+/+^;p53^−/−^ MEFs were transduced with Adeno-GFP or Adeno-shRNA Vps15 vectors. The 24-h postinfection cells were serum starved for 24 h followed by stimulation with 1 μM insulin for indicated times. Total protein extracts were analysed by immunoblotting with indicated antibodies. Immunoblot with anti-actin antibody served as a loading control.

The improved metabolic profile of *Vps15* mutants prompted us to test the therapeutic benefits of *Vps15* targeting in the models of metabolic challenge and insulin resistance. As a proof of concept, we first tested whether acute depletion of *Vps15* ameliorated glucose metabolism of HFD-challenged mice. To this end, the Vps15^f/f^ mice were subjected to short-term HFD feeding protocol and the glucose tolerance test (GTT) was performed 2 weeks later (Fig. 7a). Mice were then assigned to two experimental groups, which were injected either with CRE- or GFP-expressing adenoviral vectors. Five days postinjection mice were subjected to the GTT challenge. Unlike Adeno-GFP-treated mice, acute short-term depletion of hepatic Vps15 significantly ameliorated the glucose tolerance of HFD-fed Vps15^f/f^ mice (Fig. 7a). Next, we asked whether the metabolic improvement on hepatic *Vps15* inactivation was dependent on functional IR. To this end, we used a mouse model of inducible hepatocyte-specific depletion of IR, iLIRKO mutants. Two months after inducing the CRE-mediated recombination, iLIRKO mice developed significant glucose intolerance (Fig. 7b). Importantly, short-term *Vps15* depletion using shRNA-expressing adenoviral vectors improved glucose tolerance in control, but not in hepatic *IR* mutants (Fig. 7b). Further molecular analysis in primary hepatocyte cultures of control and iLIRKO mice revealed that loss of *IR* expression precluded activation of Akt signalling by *Vps15* downregulation (Fig. 7c). To rule out any chronic adaptation to hepatic *IR* loss, we also assayed the effect of *Vps15* downregulation in the *IR*-depleted hepatocytes by transduction with Adeno-CRE vectors (Supplementary Fig. 12). Similarly to the cultures prepared from iLIRKO mice, the acute loss of *IR* impeded the Akt stimulation by *Vps15* depletion (Supplementary Fig. 12).

To extend our observations on the effects of *Vps15* inactivation on IR/Akt signalling, we used an additional model of insulin resistance. Previous works demonstrated that loss of tumour suppressor tuberous sclerosis complex 2 (*TSC2*) gene leads to insulin resistance due to reduction in Akt phosphorylation^39^. The latter is ascribed to mTORC1-dependent negative feedback mechanisms and the defects in mTORC2 activation^39^,^40^. The expression of IRβ was strikingly downregulated in *TSC2*-null cells, suggesting additional mechanism of regulation (Fig. 7d). Importantly, unlike IGF1R, IRβ levels could be rescued by depletion of *Vps15* in *TSC2*-deficient mouse embryonic fibroblasts (MEFs) (Fig. 7d). Normalization of IRβ levels was paralleled by increase in Akt phosphorylation (Fig. 7d). Significantly, the depletion of *Vps15* in *TSC2*-null MEFs improved insulin-stimulated Akt activation and phosphorylation of its downstream target—Pras40 (Fig. 7e). In sum, the increase of Akt signalling on *Vps15* depletion is IR dependent and may rescue the defect in the model of IR resistance due to *TSC2* inactivation.

### Metabolic amelioration on acute Vps15 inactivation *in vivo*

**Figure 8:**
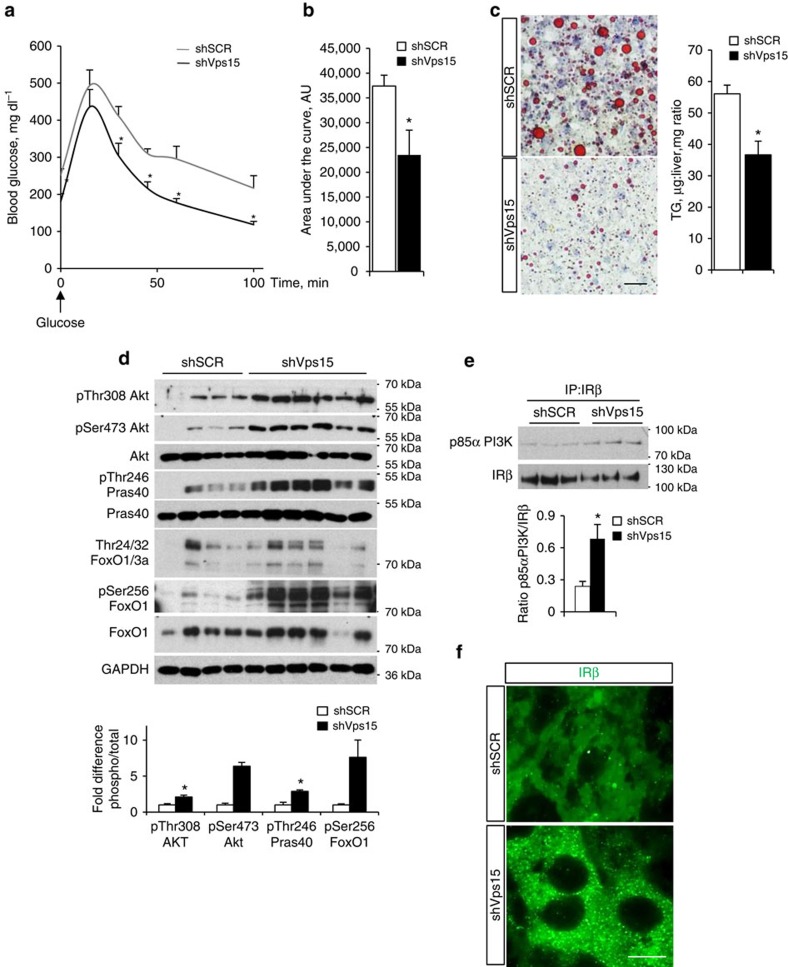
Depletion of hepatic Vps15 improves metabolic phenotype of *ob/ob* mice. (**a**) Intraperitoneal GTT in overnight-starved *ob/ob* mice 7 days post-transduction with Adeno-shRNA SCR or Adeno-shRNA Vps15 vectors. Mice were injected with 1 g kg^−1^ of glucose and glycaemia measured at indicated times. Data are means±s.e.m. (*n*=4–6, *P<0.05 versus Adeno-shRNA SCR, two-tailed, unpaired Student’s *t*-test). (**b**) The histogram of an average value of the area under the respective curve presented in **a** was calculated with GraphPadPrizm5 software. Data are means±s.e.m. (*n*=4–6, *P<0.05 versus Adeno-shRNA SCR, two-tailed, unpaired Student’s *t*-test). (**c**) Oil red staining of frozen liver sections of random-fed *ob/ob* mice killed 8 days post-transduction with Adeno-shRNA SCR or Adeno-shRNA Vps15 vectors (left panel), scale bar, 50 μm. Triglyceride (TG) content measured enzymatically in livers of random-fed *ob/ob* mice 8 days post-transduction with Adeno-shRNA SCR or Adeno-shRNA Vps15 vectors (right panel). Data are means±s.e.m. (*n*=4–6, *P<0.05 versus Adeno-shRNA SCR, two-tailed, unpaired Student’s *t*-test). (**d**) Immunoblot analyses of liver extracts from random-fed *ob/ob* mice 8 days post-transduction with Adeno-shRNA SCR or Adeno-shRNA Vps15 vectors. Densitometric analyses of phosphoprotein levels normalized to total protein levels presented as folds over shRNA SCR. Data are means±s.e.m. (*n*=4–5, *P<0.05 versus Adeno-shRNA, two-tailed, unpaired Student’s *t*-test). (**e**) Immunoblot analyses of p85αPI3K immunoprecipitated in complex with IRβ. IRβ was immunoprecipitated with anti-IRβ antibody from liver total protein extracts of random-fed *ob/ob* mice sacrificed 8 days post-transduction with Adeno-shRNA SCR or Adeno-shRNA Vps15 vectors. Densitometric analysis of p85αPI3K normalized to immunoprecipitated IRβ protein levels is presented. Data are means±s.e.m. (*n*=3–6, *P<0.05 versus Adeno-shRNA SCR). (**f**) Immunofluorescence analyses of formalin-fixed paraffin-embedded liver sections from random-fed *ob/ob* mice 8 days post-transduction with Adeno-shRNA SCR or Adeno-shRNA Vps15 vectors with anti-IRβ antibody. Secondary anti-rabbit IgG Alexa Fluor 565 antibody was used for detection (images presented in green pseudocolour). Scale bar, 10 μm.

To further test whether the *Vps15* depletion could have therapeutic benefits *in vivo* in animal models of diabetes and insulin resistance, the *ob/ob* mice were injected with adenoviral vectors expressing shRNA against Vps15. One week after injection, the body weight, liver and peripheral organ weight were not modified by the treatment (Supplementary Fig. 13a). Importantly, this treatment led to a 70% and a 50% decrease in hepatic Vps15 transcript and protein levels, respectively (Supplementary Fig. 13b,c). At the same time, the expression of Vps15 in WAT and muscle tissues was unmodified (Supplementary Fig. 13d). Downregulation of hepatic Vps15 was accompanied by impaired autophagic processing of LC3 and accumulation of p62 in the livers of shRNA Vps15-treated mice (Supplementary Fig. 13c,e). Remarkably, hepatic downregulation of *Vps15* significantly improved glucose tolerance of *ob/ob* mice (Fig. 8a,b). This was accompanied by a decrease in liver steatosis on *Vps15* depletion (Fig. 8c). The plasma metabolite levels were not modified by the treatment (Supplementary Fig. 14a). The decrease in hepatic triglyceride levels was accompanied by reduction of lipogenic transcription activators and lipogenic enzyme expression (Supplementary Fig. 14b,c). The expression of the lipases was induced in the livers of *ob/ob* mice on *Vps15* depletion suggesting the induction of lipolysis (Supplementary Fig. 14b). Expression of PGC1α transcriptional coactivator was significantly decreased (Supplementary Fig. 14b). The expression of glycolytic enzymes HK2 and PKM2 was markedly induced (Supplementary Fig. 14c). Similarly, hepatic *Vps15* downregulation reverted glucose intolerance in wild-type mice fed for short term with HFD (Supplementary Fig. 14d). Altogether, the acute depletion of *Vps15* in livers improves the metabolic parameters in genetic and diet-induced animal models.

### *Vps15* depletion improves hepatic IR signalling in *ob/ob* mice

In line with our earlier observations, shRNA silencing of hepatic *Vps15* in obese mice resulted in a sharp activation of Akt as evidenced by Ser473 and Thr308 phosphorylation (Fig. 8d). The activation of the pathway was further corroborated by a significant increase in the phosphorylation of Akt substrates—Pras40 and FoxO1 (Fig. 8d). The phosphorylation of Akt substrates was not modified in muscles and fat tissue of treated mice (Supplementary Fig. 13d). In addition, immunoprecipitation analyses with anti-IRβ antibody revealed that p85αPI3K was more abundant in the complexes precipitated from the extracts of livers of shRNA Vps15-treated mice as compared with controls (Fig. 8e). Finally, we asked whether IR localization is affected in shRNA Vps15-transduced mice. Microscopic examination of liver sections of shVps15-treated mice revealed marked increase in IRβ-positive endosomes (Fig. 8f). Taken together, our data suggest that selective inhibition of hepatic class III PI3K may have beneficial consequences in conditions of T2D and metabolic syndrome, by ameliorating glucose metabolism through improved IR signalling.

## Discussion

In this study we provide experimental evidence that class III PI3K is regulated by insulin in hepatocytes and in turn modulates IR signalling to insure whole-body metabolic responses. We show that UVRAG-associated class III PI3K interacts with IR and is activated by insulin, with kinetics that are consistent with a role in IR endocytosis and trafficking. Interference with the expression of the regulatory subunit in the class III PI3K complex, Vps15, results in three novel and yet unreported outcomes: (i) defects of IR degradation and increased Akt signalling accompanied by profound perturbations in endocytic trafficking; (ii) blunted gluconeogenesis concomitant with nuclear exclusion of FoxO1 transcription factor and defects in FoxO1-dependent gene expression; and (iii) amelioration of metabolic syndromes in genetic and diet-induced animal models. These data reveal a previously unappreciated role of class III PI3K in the control of insulin sensitivity and metabolic adaptations.

How class III PI3K activity is regulated by extracellular signals has been a long-standing question in the field. Insulin, glucose and amino-acid levels that effectively modulate endocytosis and autophagy, have deceivingly modest effects on total Vps34 lipid kinase activity^22^,^24^,^41^. An explanation has recently came from the demonstration that glucose and amino-acid starvation stimulate specific Vps34-containing complexes without having a significant effect on the total Vps34 activity in some cells^22^,^23^. However, the effect of insulin on specific complexes has never been tested. In the present study we show that, while amino-acid starvation potently induces ATG14-containing class III PI3K activity, insulin stimulation selectively promotes the activity of UVRAG-containing class III PI3K (Fig. 3). Importantly, this evidence provides a regulatory mechanism for the reported functions of these distinct complexes, that is, the induction of autophagy during nutrient starvation by the ATG14-containing complex and the promotion of endocytic trafficking during growth factor stimulation by the UVRAG-containing complex. How insulin specifically stimulates UVRAG-containing complexes will require further investigation. Interestingly, we show that IR is found in complex with class III PI3K (Fig. 3c,d and Supplementary Fig. 3c). Bioinformatic analysis by online resource ScanSite3 (ref. 42) suggests the presence in UVRAG and Vps15 of putative SH2-binding domains that recognize phospho-tyrosine. In addition, both UVRAG and Vps15 are identified as tyrosine phosphorylated proteins in independent phosphoproteome studies including those accessible in Phosphosite database^43^. It is therefore possible a direct action of IR/IRS on UVRAG-containing class III PI3K complexes, similar to the known effect on class I PI3K. Consistently, another component of class III PI3K complex, Beclin-1, has been recently reported to be tyrosine phosphorylated by epidermal growth factor receptor, resulting in inhibition of Vps34 activity^44^. However, we cannot exclude intermediate steps, possibly involving the Rab family of small GTPases, which are also actively involved in endosomal trafficking and found in complex with class III PI3K^45^,^46^,^47^. Of note, Rab5 has been shown to co-localize with IR in endosomes and its GTP loading is potently stimulated by insulin^48^. In addition, Rab7 can interact with class III PI3K complexes and stimulate its activity^45^,^49^. Therefore, the crosstalk between IR-Rab5/Rab7-Vps34/Vps15/UVRAG is likely to exist at distinct levels in insulin-stimulated cells.

We demonstrate that depletion of *Vps15* increases insulin sensitivity in different models both *in vitro* and *in vivo* (Figs 1, 4, 7 and 8). This suggests that Vps34/Vps15 complex is involved in a feedback regulatory loop that, on insulin stimulation, contributes to the modulation of IR function. The effects of class III PI3K inactivation are complex. At current stage, it is difficult to separate the general trafficking defect due to PI3P depletion from potential novel direct mechanisms of class III PI3K on IR function. However, it is likely that the IR-signalling perturbations we observed on *Vps15* depletion are due to compromised endocytic trafficking of the receptor. The reduced velocity of IR-positive endosomes (Fig. 2b,c and Supplementary Movie) together with the striking reorganization of the endosomal compartment (Figs 1d and 2a and Supplementary Figs 1d, 2d and 6g) suggest that trafficking defects might largely contribute to the delayed receptor degradation. In these conditions, prolonged residence of IR in endosomes would contribute to the amplified insulin signalling that we measured. Indeed, key insulin signal transduction elements are found at endosomal membranes including IRS, class I PI3K and Akt^7^. Accordingly, preventing IR degradation by blocking its intracellular transport resulted in similar increase in insulin-dependent signalling (Fig. 2d). Of note, an endosomal protein, APPL1, has been reported to favour downstream insulin signalling through the Akt pathway^50^,^51^. APPL1 was identified in protein interaction screens as a binding partner of Akt and an adaptor protein positively regulating Akt activity^52^,^53^,^54^. It has been shown to favour the recruitment of IRS proteins to the IR and to release Akt from the binding to TRB3, its endogenous inhibitor^50^,^51^. Consistently, *in vivo* studies using gain- or loss-of-function approaches have confirmed the positive role of APPL1 on insulin sensitivity^50^,^51^. Interestingly, PI3P is required for the maturation of APPL1-positive endosomes to sorting endosomes^54^. It would be interesting to test whether loss of functional class III PI3K affects APPL1^+^ endosomes contributing to the observed metabolic responses.

In addition, it is plausible that the endosomal sorting and lysosomal degrading activities are lower in *Vps15*-depleted livers, partly accounting for the slower kinetics of IR downregulation after insulin stimulation. In a previous study on skeletal muscles, we have shown that the lysosomal function is affected by the *Vps15* depletion due to sorting defects of proteolytic enzymes^26^. Of note, chloroquine, a drug that interferes with the acidification of the endosomes, prevents dissociation of insulin from its receptor and subsequent receptor degradation, also potentiates insulin action and has beneficial effects on glucose homeostasis^6^,^55^,^56^,^57^.

Massive vacuolation is a characteristic morphological change in the cells depleted of *Vps15* apparent both *in vitro* and *in vivo*. Despite highly resembling the steatosis, the histological analyses by Oil Red and Bodipy staining did not reveal apparent accumulation of neutral lipids in hepatic tissue neither on acute or chronic *Vps15* depletion. Furthermore, the acute *Vps15* downregulation in the livers of obese mice decreased triglyceride levels. At first sight, those are unexpected observations as hepatic inactivation of essential autophagic genes, *Atg5* and *Atg7*, was reported to induce steatosis due to defective lipophagy^14^,^31^,^32^. However, we cannot exclude that other types of lipids that could not be revealed by the analyses performed (for example, polar lipids, including sphingolipids, phospholipids and ceramides) accumulate on *Vps15* depletion. This is in line with the observations of increased phospholipid content on chronic chloroquine treatment, which provoke cytoplasmic vacuolation in different cell types including hepatocytes^58^. In addition, the vacuolation of hepatocytes could be a morphological manifestation of cytoplasmic accumulations of various substances, including water and glycogen, all having similar morphology. Those structures could originate from different membranes including plasma membrane, endosomal compartment, Golgi apparatus, lysosomes or autophagolysosomes at the different stages of the maturation. The subcellular origin and the content of abundant vacuolar structures in *Vps15*-depleted cells require further studies. From the other hand, the relationship between defective autophagy and the steatosis as well as steatosis and insulin resistance are not completely understood. Of note, increased insulin sensitivity of hepatocyte-specific *PTEN* mouse mutants is accompanied by severe steatosis and defective autophagy^59^,^60^. In contrast, hepatic mutants of *TSC1*, which are characterized by severe autophagy block and insulin resistance, are protected from diet-induced steatosis^61^,^62^. Considering that class III PI3K controls both autophagy and endocytic trafficking, the effects of its inactivation will be manifested on multiple levels.

The role of FoxO1 transcription factor as a chief regulator of gluconeogenic responses in the liver is firmly established^33^,^34^,^35^,^63^,^64^. In addition, interference with FoxO1 activity or expression in mouse models of obesity or insulin resistance significantly improves glucose tolerance^35^,^63^,^64^. These observations are highly reminiscent of the Vps15 LKO mouse phenotype (Figs 4, 5, 7 and 8 and Supplementary Figs 7 and 14). Similarly to therapy with Foxo1 antisense oligonucleotides in DIO mice^35^, depletion of *Vps15* in *ob/ob* mice improves steatosis and glucose metabolism (Fig. 8a–c). The best documented point of control of FoxO1 is Akt-dependent phosphorylation and inactivation in response to insulin^36^. In line with blunted gluconeogenesis observed in *Vps15*-depleted hepatocytes and *in vivo*, we detect increased phosphorylation of FoxO1 on *Vps15* inactivation. Our observations in Vps15 LKO mice (Figs 4, 5, 6) are in agreement with the established mechanism of IR/Akt-dependent FoxO1 phosphorylation, leading to nuclear exclusion and proteasomal degradation^37^,^38^. However, we cannot rule out the possibility that the striking suppression of FoxO1 function in *Vps15*-depleted hepatocytes is also the consequence of a direct action of class III PI3K on FoxO1.

The central role of endocytic and autophagic trafficking in nutrient/growth factor sensing and homeostasis has prompted numerous studies on the metabolic consequences of their functional alterations. The physiological importance of autophagy for the maintenance of glycaemia during the perinatal period before lactation is illustrated by the perinatal lethality of the mouse autophagy mutants^31^,^65^. Defective autophagy is discussed both as a causative factor as well as accompanying state in many pathological conditions^10^. Mouse models of obesity display autophagy defects and the metabolic syndrome could be improved by increasing autophagic flux through Atg7 overexpression or by autophagy enhancers^14^,^15^. However, a defect in autophagic flux in skeletal muscles or liver by *Atg7* deletion may also lead to increased circulating levels of Fgf21 and protect from the detrimental effects of HFD feeding^13^.

Here we show that *in vivo* hepatic depletion of *Vps15* has strikingly different outcomes on whole-body nutrient homeostasis as compared with autophagy mutants. Despite the involvement of class III PI3K in autophagy, the metabolic effects of hepatic *Vps15* depletion can be ascribed to a prominent role on IR endocytic trafficking and signal transduction through the alteration of a retrograde signalling mechanism. Defects of IR endocytosis trafficking and degradation are reported in metabolic conditions accompanied by hyperinsulinemia, such as genetic forms of obesity and T2D^11^,^12^,^66^. Our data reveal the impact of altering Vps34/Vps15 function in metabolic syndromes and suggest a novel therapeutic target in these diseases.

## Methods

### Reagents

The following primary antibodies were used: Vps15 (1:1,000, Abnova, H00030849-M03; or 1:1,000, Genetex, GTX108953); p62 (SQSTM) (1:3,000, Abnova, H00008878-M01); Tubulin (1:5,000, Sigma, T9026); β-actin (1:50,000, Sigma, A5316); Beclin-1 (1:1,000, Cell Signaling, 3495); pSer473 Akt (1:1,000, Cell Signaling, 9275); pThr308 Akt (1:1,000, Cell Signaling, 9275); Akt (1:1,000, Cell Signaling, 9272); pThr246 Pras40 (1:1,000, Cell Signaling, 2997); Pras40 (1:1,000, Cell Signaling, 2691); GAPDH (1:1,000, Santa Cruz, SC-25778); pY IRβ (1:1,000, Cell Signaling, 3024 and 3021); Rab5 (1:1,000, Cell Signaling, 3547); Rubicon (1:1,000, Cell Signaling, 8465); pThr24/32 FoxO1/3a (1:1,000, Cell Signaling, 9464); pSer256 FoxO1 (1:1,000, Cell Signaling, 9461); FoxO1 (1:1,000, Cell Signaling, 2880); Lamin A/C (1:1,000, Cell Signaling, 2032); GST (1:5,000, Santa Cruz, SC-459); IGF1R (1:1,000, Cell Signaling, 9750); p85α PI3K (1:1,000, Santa Cruz, SC-423); IRS1 (1:500, Millipore, 06-248); UVRAG (1:1,000, Cell Signaling, 13115); Atg14 (1:1,000, Cell Signaling, 5504); Vps34 (1:1,000, Cell Signaling, 4263); LC3 (1:1,000, NanoTools, 0231-100/LC3-3-5-5F10); Ub (1:1,000, Cell Signaling, 3963); FAS (1:1,000, Cell Signaling, 3180); ATPCL (1:1,000, Cell Signaling, 4332); HK2 (1:1,000, Cell Signaling, 2867); PKM2 (1:1,000, Cell Signaling, 4053); Enolase (1:1,000, Santa Cruz, SC-7455); PPARγ (1:1,000, Cell Signaling, 2435); ACC (1:1,000, Cell Signaling, 3662); Flag-tag (1:1,000, Sigma, F3162); LAMP1 (1:1,000, Abcam, ab24170); LAMP2 (1:1,000, Abcam, ab13524); BrdU (1:500, Roche, 11170376001); β-catenin (1:500, BD Biosciences, 610153); VDAC (1:1,000, Calbiochem, AB10527); G6PC (1:1,000, Santa Cruz, SC-33839 and a kind gift of Gilles Mithieux (INSERM U855, France)); PEPCK (1:1,000, Santa Cruz, SC-32879); and IRβ (1:1,000, Santa Cruz, SC-711). For class III PI3K *in vitro* activity antibodies were from MBL International ATG14 (PD026) and UVRAG (M160-3). Vps34 antibody used for class III PI3K *in vitro* activity was from Echelon Biosciences (Z-R015). GFP and GFP-CRE adenoviral vectors were described previously^26^. Adenoviral vector expressing shRNA Vps15 was generated and amplified by VectorBiolabs (USA). Adenovirus expressing shRNA Scrambled was kindly provided by Stephan Herzig (Institute for Diabetes and Cancer, Germany). 2xFYVE-GFP-expressing vector was kindly provided by Sharon Tooze (CRUK, UK). hIRβ-RFP-overexpressing vector was kindly provided by Ingo B. Leibiger (Karolinska Institutet, Sweden).

### Animals

The Vsp15 conditional mutant mouse line was established at the MCI/ICS (Mouse Clinical Institute—Institute Clinique de la Souris, Illkirch, France) as described in ref. 26. For generation of liver-specific *Vps15* knockout mouse line, *Vps15* floxed mice were crossed with transgenic mice expressing Cre recombinase under the control of a Rat albumin promoter^30^. For the genotyping genomic DNA isolated from mouse tail snip or tissues was analysed by PCR. The primer sequences are listed in Supplementary Table S1. The following combinations of the primers were used: to check presence of distal loxP sites—LF/LR; to check Cre-mediated excision of the locus—LF/ER. For generation of liver-specific IR knockout mouse line, mice carrying the LoxP sites flanking the fourth exon of the *IR* gene (IR^lox/lox^ stock number: 006955; Jackson laboratory, USA) were intercrossed with C57BL/6J, which specifically express the Cre recombinase in the liver under the transthyretin promoter (TTR-Cre^Tam^ mice; kind gift of Mireille Vasseur, Institut Cochin, France)^67^. The resulting IR^lox/+;TTR-CreTam^ mice were interbred with IR^lox/lox^ mice to generate IR^lox/lox;TTR-CreTam^ mice, named iLIRKO (for inducible liver insulin receptor knockout). To knockout the *IR* gene, 12-week-old male mice were submitted to an intraperitoneal tamoxifen injection (1.5 mg per mouse) during three consecutive days. The efficient deletion of IR specifically in hepatocytes was observed 2 weeks postinjection. iLIRKO mice 8 weeks postinjection with the tamoxifen were used for the *in vivo* experiments. Five-week-old male *ob/ob* and C57Black/6 mice were purchased from Janvier (France). Mice were acclimatized for 2 weeks in the animal facility before the initiation of the treatments. Male mice were used for the experimentation. All animal studies were approved by the Direction Départementale des Services Vétérinaires, Préfecture de Police, Paris, France (authorization number 75-1313).

### Treatments and metabolic studies *in vivo*

All animals used in the study were fed *ad libitum* standard chow diet (Teklad global protein diet; 20% protein, 75% carbohydrate and 5% fat). Where indicated, mice were fed with HFD (Diet D12492(I) SNIFF Diet; 20% protein, 20% carbohydrate and 60% fat) for 2 weeks before manipulations. For 5-bromo-2′deoxyuridine (BrdU) incorporation, mice were treated with BrdU (3 mg ml^−1^, Sigma-Aldrich) dissolved in drinking water for 3 days before killing. Animals were killed between 14:00 and 16:00 unless indicated. For immunohistochemical analysis liver tissue was fixed overnight in phosphate-buffered 10% formalin and embedded in paraffin. In all, 6-μm sections were cut and processed either for staining with eosin/hematoxylin or for immunohistochemical analyses. Glucose and pyruvate tolerance test were performed in 6-week-old Vps15 LKO mice after an overnight fasting (14 h). Insulin tolerance test was performed on 6-h starved 6-week-old control and Vps15 LKO mice. Mice were injected intraperitoneally with glucose (Vps15^f/f^, Vps15 LKO, iLIRKO, chow-fed wild-type and HFD-fed wild-type mice—2 g kg^−1^, *ob/ob*—1 g kg^−1^), Humalog insulin (1 μ kg^−1^) or pyruvate (2 g kg^−1^), and blood was collected from the tail vein for determination of glucose levels at different time points using Glucotrend glucometer (Roche Diagnostics). Triglyceride levels in the acetone extracts of liver tissue were determined using Triglycerides FS Kit (Diasys) according to the manufacturer’s instructions and as described^68^.

### Cell culture

Mouse hepatoma cell line Hepa1.6 was obtained from American Type Culture Collection. Immmortalized TSC2^−/−^;p53^−/−^ MEFs and the littermate-derived pair wild-type control cells were provided by D. J. Kwiatkowski (Brigham and Women’s Hospital, Boston, MA) and were described previously^69^. Cells were maintained in Dulbecco’s modified Eagle’s medium supplemented with 10% foetal bovine serum (FBS), 2 mM L-glutamine, 50 U ml^−1^ penicillin and 50 μg ml^−1^ streptomycin. To achieve Vps15 depletion, Hepa1.6 cells and MEFs were infected with 50 m.o.i. (multiplicity of infection) of Adeno-shRNA Vps15 or as a control with Adeno shRNA SCR or GFP-expressing vectors. Cells were collected 48 h later for the analyses. For insulin treatment, 24 h postinfection cells were starved in Dulbecco’s modified Eagle’s medium without FBS for 24 h and then were stimulated with insulin (final concentration 1 μM) for indicated times.

Primary hepatocytes from 4–6-week-old mice were isolated by liver perfusion as described previously^68^. Hepatocytes were plated at 12 × 10^4^ cells per cm^2^ in Williams medium (Life Technologies) supplemented with 20% FBS, 100 nM Insulin, 25 nM dexamethasone, penicillin (100 U ml^−1^), streptomycin (100 μg ml^−1^) and amphotericin B (Fungizone; 250 ng ml^−1^). To achieve *Vps15* deletion Vps15^f/f^ hepatocytes 12 h after plating were infected with 10 m.o.i. of Adeno-CRE or as a control with Adeno-GFP vectors, 2 h after addition of viral particles the media was changed to Williams medium supplemented with mix of antibiotics and 25 nM dexamethasone, and when indicated 10% FBS and 100 nM insulin were added. Cells were collected 72 h later for the analyses. For insulin treatment, hepatocytes 72 h postinfection were stimulated with 1 μM insulin for different times.

### Glucose production in primary hepatocytes

Primary hepatocytes were plated in 12-well plates. The 12-h post-platting cells were infected with Adeno-shRNA SCR or Adeno-shRNA Vps15 vectors. The 48-h postinfection cells were washed once with PBS, and glucose production was determined after 12 h of incubation in Krebs salts solution supplemented with lactate/pyruvate mix (10:1 mM). The amount of glucose released into the medium was determined by evaluating the production of NADPH from NADP in the presence of hexokinase and glucose-6-phosphate dehydrogenase using commercially available kit (Sigma) and normalized to the total protein content in each well determined by Bradford assay (BioRad).

### Subcellular fractionation and endosome preparation

Nuclear extracts were prepared using NE-PER Kit (Pierce) according to the manufacturer’s recommendations, from 1 × 10^6^ cells. Endosomal fraction was purified using sucrose step gradient as described^70^. Briefly, cells were washed with PBS and were pelleted by centrifugation at 200*g* for 5 min. The equal mass of cells was used for each condition. The pellet was resuspended in homogenization buffer (HB: 250 mM sucrose (8.6%), 3 mM imidazole (pH 7.4), supplemented with protease inhibitors (Roche)) and was homogenized by seven passes through a G25 needle. Post-nuclear supernatant (PNS) was obtained by centrifugation at 2,000*g* for 10 min. Then, sucrose concentration of the PNS was adjusted to 40.6% and PNS was transferred to SW41 ultracentrifuge tube (Beckman-Coulter). Next, 35% sucrose in 3 mM imidazole buffer (pH 7.4) was layered on PNS up to the top of the tube. The sample was centrifuged at 210,000*g* for 3 h at 4 °C. Crude endosomes were collected from the interphase between 35% sucrose and HB buffer.

### Microscopy

For fluorescent microscopy analyses, hepatocytes were grown on collagen-treated coverslips (Millipore). The 12-h post-plating primary hepatocytes were transfected with corresponding plasmids using Lipofectamine 2000 reagent as recommended by the manufacturer (Life Technologies). For the microscopy analyses 24 h post-transfection cells were fixed with 4% paraformaldehyde in PBS for 20 min and permeabilized with 0.1% saponin in PBS for 10 min, followed by blocking in 3% BSA in PBS. Slides were then treated with primary antibodies for 1 h or directly analysed by fluorescent microscopy. Secondary antibodies used for these assays were anti-rat IgG Alexa Fluor 635 (Life Technologies) or anti-rabbit IgG Alexa Fluor 488 or 565 (Life Technologies). Confocal images were acquired with an optical slice of 0.8 μm using a × 40/0.75 oil immersion objective using LSM 700 confocal microscope (Zeiss) and analysed using ZEN software (Zeiss). Fluorescence and light microscopy were performed using an inverted microscope (Eclipse Ti-S; Nikon) and × 10/0.30, × 20/0.50 or × 40/0.785 Plan Fluor objectives (Nikon). Images were captured using a Super high-definition cooled colour camera head DS-Ri1 (Nikon) and NIS Elements software (Nikon). All samples for microscopy were viewed at room temperature.

For spinning disk microscopy, cells plated onto glass-bottom dishes and transfected with the indicated constructs were imaged for exposure times of 200 ms at 5-s intervals for 240 s using a spinning disk microscope (Andor) based on a CSU-W1 Yokogawa head mounted on the lateral port of an inverted IX-83 Olympus microscope equipped with a × 60 objective lens and a 491 nm 100 mW laser (Andor) used at 40% of maximum power. Images were acquired with a sCMOS camera (Andor). The system was steered by Metamorph 7 software.

### Protein extraction, immunoblotting and immunoprecipitation

To prepare protein extract for immunoblot analysis, cells were washed twice with cold PBS, scraped from the dishes in lysis buffer containing 20 mM Tris-HCl (pH 8.0), 5% glycerol, 138 mM NaCl, 2.7 mM KCl, 1% NP-40, 20 mM NaF, 5 mM EDTA, 1 × protease inhibitors (Roche) and 1 × PhosphoStop Inhibitors (Roche). The same buffer was used to prepare protein extracts from liver tissue. Homogenates were spun at 12,000*g* for 10 min at 4 °C. For immunoprecipitation 1 mg of cleared protein extract was incubated with 1 μg of anti-Flag M2 (Sigma), anti-IRβ antibody (Santa Cruz), anti-UVRAG (MBL) or anti-p85αPI3K antibody (Santa Cruz) for 2 h at 4 °C. Then, immune complexes were pulled down using Protein G Sepharose beads (GE) during 1 h followed by four washes with extraction buffer. Protein extracts or immunoprecipitates were resolved by SDS–polyacrylamide gel electrophoresis before transfer onto PVDF membrane followed by incubation with the primary antibodies and horseradish peroxidase-linked secondary antibodies. Immobilon Western Chemiluminescent HRP Substrate (Millipore) was used for the detection.

### *In vitro* Vps34 lipid kinase assay

Cells were lysed in Mild Lysis Buffer (MLB: 10 mM Tris, pH7.5, 2 mM EDTA, 100 mM NaCl, 1% NP-40, 50 mM NaF, 1 mM Na3VO4 and protease inhibitor cocktail (Roche)). Total protein extracts were cleared by 10-min centrifugation at 11,000*g*. For immunoprecipitation, the indicated antibody was coupled with protein G Sepharose (GE). The antibody-conjugated beads were added to the cell lysates and incubated at 4 °C for 2 h. Beads were washed with MLB four times and one time with kinase buffer (KB: 20 mM HEPES (pH 7.4), 1 mM EGTA, 0.4 mM EDTA and 5 mM MgCl_2_). One-half volume was then taken for input, the remainder was centrifuged and excess 1 × KB was removed. A volume of 40 μl of 1 × kinase assay buffer was added to the beads supplemented with 0.1 mg ml^−1^ phosphatidylinositol, 50 μM cold ATP, 5 μCi 32 P-ATP, 5 mM MnCl_2_ and 50 μM dithiothreitol followed by incubation at 37 °C for 30 min with vigorous shaking. Reaction was quenched by addition of 10 μl 1 M HCl, followed by lipid extraction with 2 volumes of MeOH:CHCl_3_ (1:1). Aqueous phase was discarded and organic phase loaded on a thin layer chromatography plate (Whatman). Resolution of phospholipid was achieved using a buffer composition of CHCl:MeOH (99%):NH_4_OH (30%):Water (129:100:4.29:24). Resolved plates were analysed by autoradiography.

### Real-time quantitative PCR

Total RNA was isolated from tissue by RNAeasy Lipid Tissue Mini Kit (Qiagen) and RNeasy Mini Kit (Qiagen) from primary hepatocytes. Single-strand complementary DNA was synthesized from 1 μg of total RNA using 125 ng of random hexamer primers and SuperScript II (Life Technologies). Real-time quantitative PCR was performed on MX3005P instrument (Agilent) using a Brilliant III Ultra-Fast QPCR Master Mix (Agilent). The relative amounts of the mRNAs studied were determined by means of the 2^−ΔΔCT^ method, with pinin or cyclophilin as reference genes and control treatment or control genotype as the invariant control. The primer sequences are listed in Supplementary Table S1.

### Statistical analysis

A two-tailed Student’s *t*-test was used for statistical analysis unless indicated. All data are expressed as means±s.e.m., and significance was established at the *P*≤0.05 level.

## Additional information

**How to cite this article:** Nemazanyy, I. *et al*. Class III PI3K regulates organismal glucose homeostasis by providing negative feedback on hepatic insulin signalling. *Nat. Commun.* 6:8283 doi: 10.1038/ncomms9283 (2015).

## Supplementary Material

Supplementary Information Supplementary Figures 1-15 and Supplementary Table 1.

Supplementary Movie 1 Time-lapse imaging of IRβ-RFP protein in Hepa1.6 cells depleted of Vps15.
