# Supplementary material for: Class III PI3K regulates organismal glucose homeostasis by providing negative feedback on hepatic insulin signalling
Source: Nat Commun. 2015 Sep 21;6:8283. doi: 10.1038/ncomms9283 (PMC4579570; doi:10.1038/ncomms9283)
Supplement: Supplementary Information — Supplementary Figures 1-15 and Supplementary Table 1. [file ncomms9283-s1.pdf]

Supplementary Figure 1

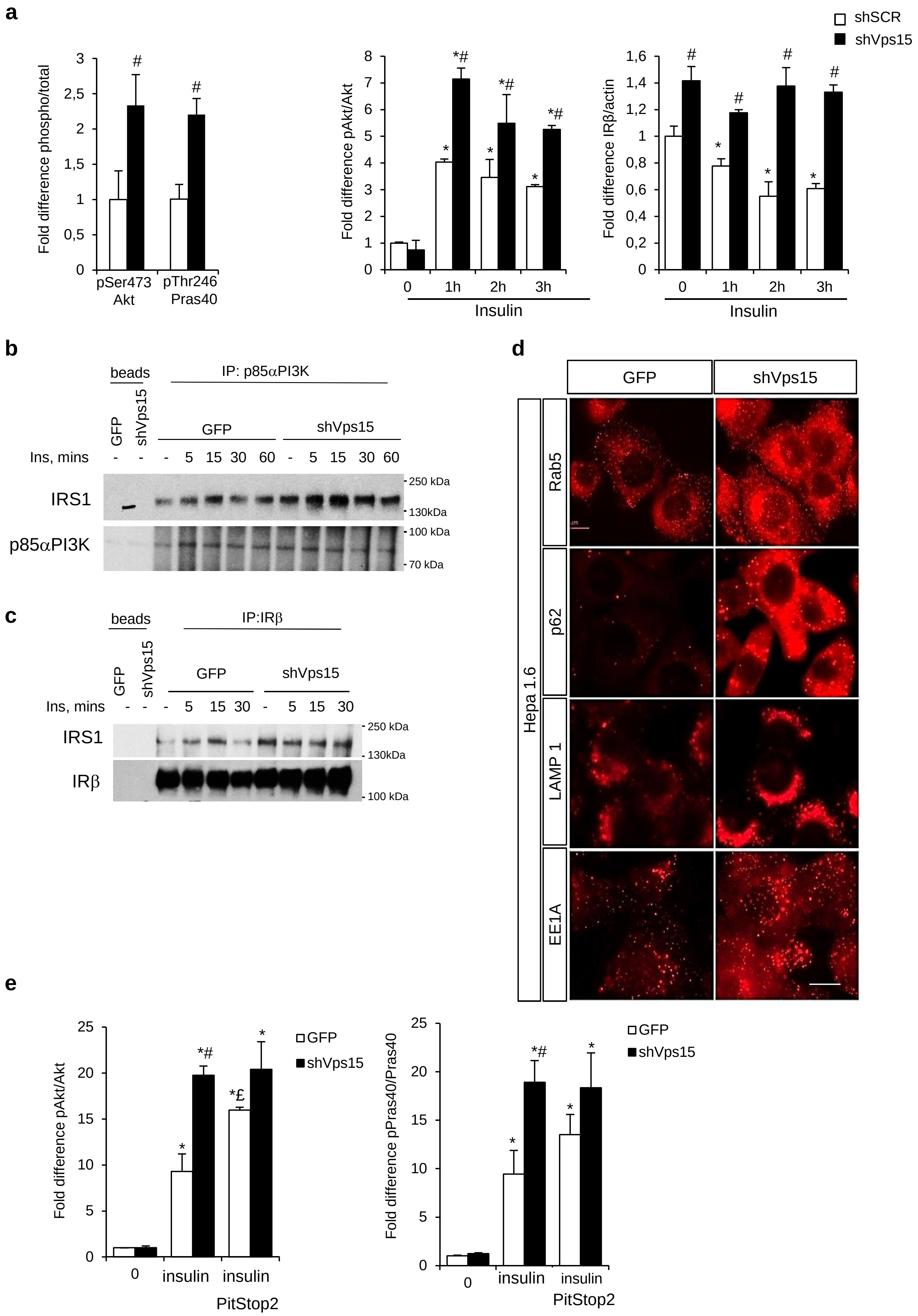

Supplementary Figure 1. Depletion of Vps15 in Hepa1.6 cells results in improved IR downstream signalling.

a. Densitometric analyses of immunoblots presented on main Fig. 1a. and Fig. 1b. IRβ protein levels, phosphorylated Akt and Pras40 were normalised to actin or total protein levels, respectively. Data are presented as fold difference over control. Data are means ±SEM (n=3-6, $P<0.05$ *: vs unstimulated, #: vs shSCR, 2-tailed, unpaired Student's t test). Immunoblot analyses of anti-p85aPI3K (**b**) and anti-IRβ immunoprecipitates (**c**) showing increased association of downstream signalling components in cells depleted of Vps15. Hepa1.6 cells were transduced with shRNA Vps15 or GFP expressing adenoviruses. 24 hours post-infection cells were serum starved for additional 24 hours followed by stimulation with 1µM insulin for indicated times. Equal amounts of total protein extracts were used for each immunoprecipitation. Co-immunoprecipitated proteins were revealed with respective antibodies. **d.** Acute depletion of Vps15 results in trafficking defects. Hepa1.6 cells were transduced with shRNA Vps15 or shRNA SCR expressing adenoviruses. 36 hours post-infection cells were PFA fixed and Rab5, Lamp1, EEA1 positive compartments (endosomes) as well as p62 positive compartment were revealed by indirect fluoresce using respective antibodies. Secondary Alexa Fluor 635 antibody was used for detection (images presented in red pseudocolor). Scale bar: 10 µm. **e.** Densitometric analyses of phosphorylated proteins normalised to total protein levels of immunoblot analyses presented on main Fig. 2d. Data are means ±SEM (n=3, $P<0.05$ *: vs unstimulated, #: vs GFP, £: vs insulin stimulated, 2-tailed, unpaired Student's t test).

Supplementary Figure 2

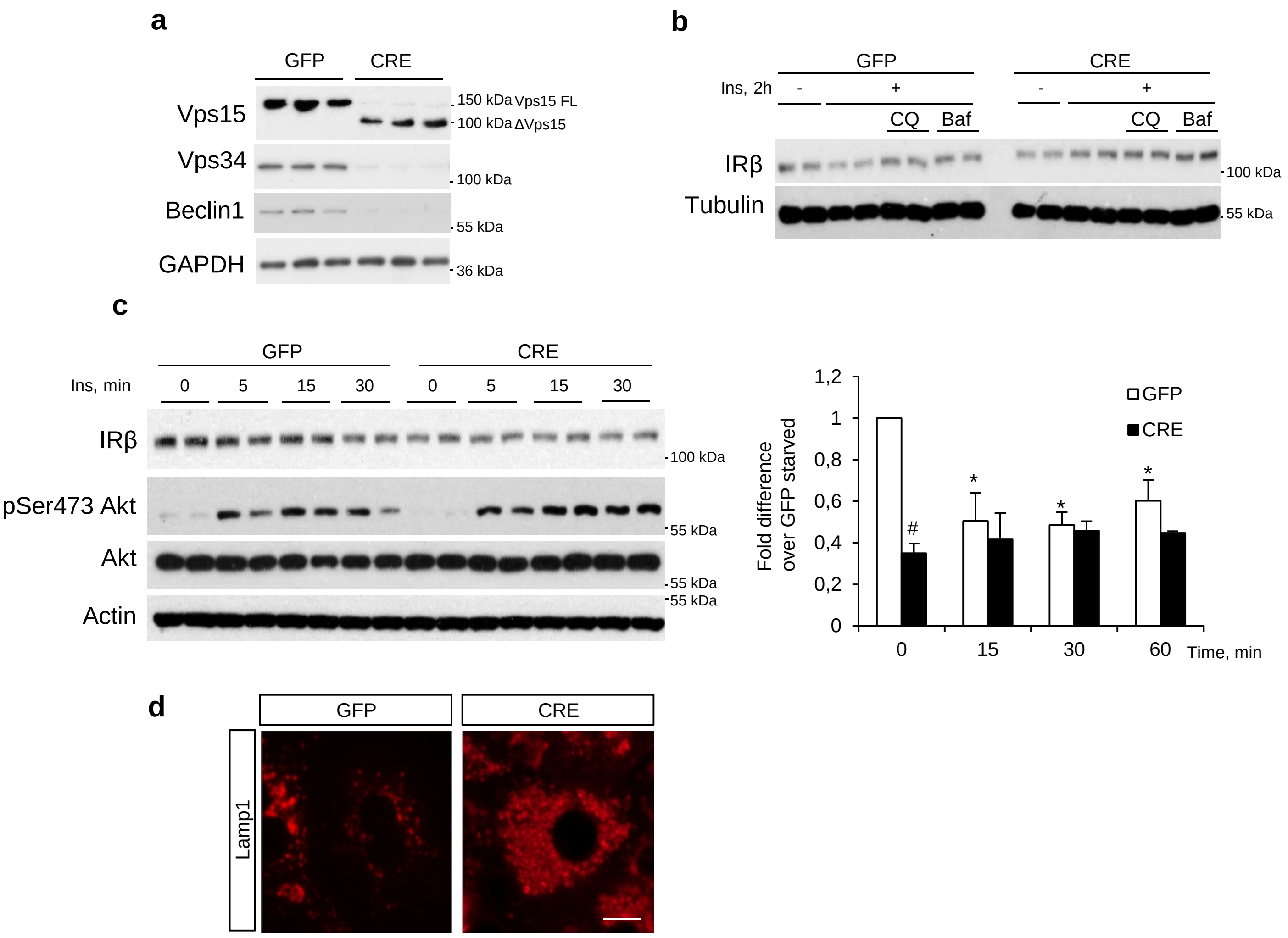

Supplementary Figure 2. Lysosomal degradation of IR is defective in *Vps15*-depleted hepatocytes.

a. Depletion of *Vps15* in primary hepatocytes results in loss of components of class III PI3K complex. Immunoblot analysis with indicated antibodies of control and *Vps15* -depleted hepatocytes 72 hours post-infection with Adeno-GFP or Adeno-CRE vectors. Immunoblot with anti-GAPDH antibody served as a loading control. **b.** Insulin stimulated IRβ degradation is dependent on lysosomal function. Vps15$^{f/f}$ primary hepatocytes were transduced with Adeno-GFP or Adeno-CRE vectors. 72 hours post-transduction serum starved cells were pre-treated with lysosomal inhibitors (chloroquine and Bafilomycin A) for 30 minutes followed by stimulation with 1µM insulin for 1 hour. IRβ protein levels were monitored by immunoblotting. Probing with anti-tubulin antibody served as loading control. **c.** Impaired degradation of IRβ in *Vps15*-depleted hepatocytes is paralleled by increased Akt phosphorylation. Vps15$^{f/f}$ primary hepatocytes were transduced with Adeno-GFP or Adeno-CRE vectors. 72 hours post-transduction serum starved cells were stimulated with 1µM insulin for indicated times. Densitometric analysis of normalised IRβ protein levels in starved and insulin-stimulated control or *Vps15*-depleted primary hepatocytes is presented. Data are means ±SEM (n=3-6, P<0.05 #: vs Adeno-GFP transduced, *: vs unstimulated, 2-tailed, unpaired Student's t test). **d.** Depletion of *Vps15* results in expansion of Lamp1 positive compartment. Primary hepatocytes were transduced with Adeno-GFP or Adeno-CRE expressing vectors. 60 hours post-infection hepatocytes were PFA fixed and Lamp1 positive compartments (endosomes) were revealed by indirect fluorescence using anti-Lamp1 antibody. Secondary anti-rat IgG Alexa Fluor 635 antibody was used for detection (images presented in red pseudocolor). Scale bar: 10 µm.

Supplementary Figure 3

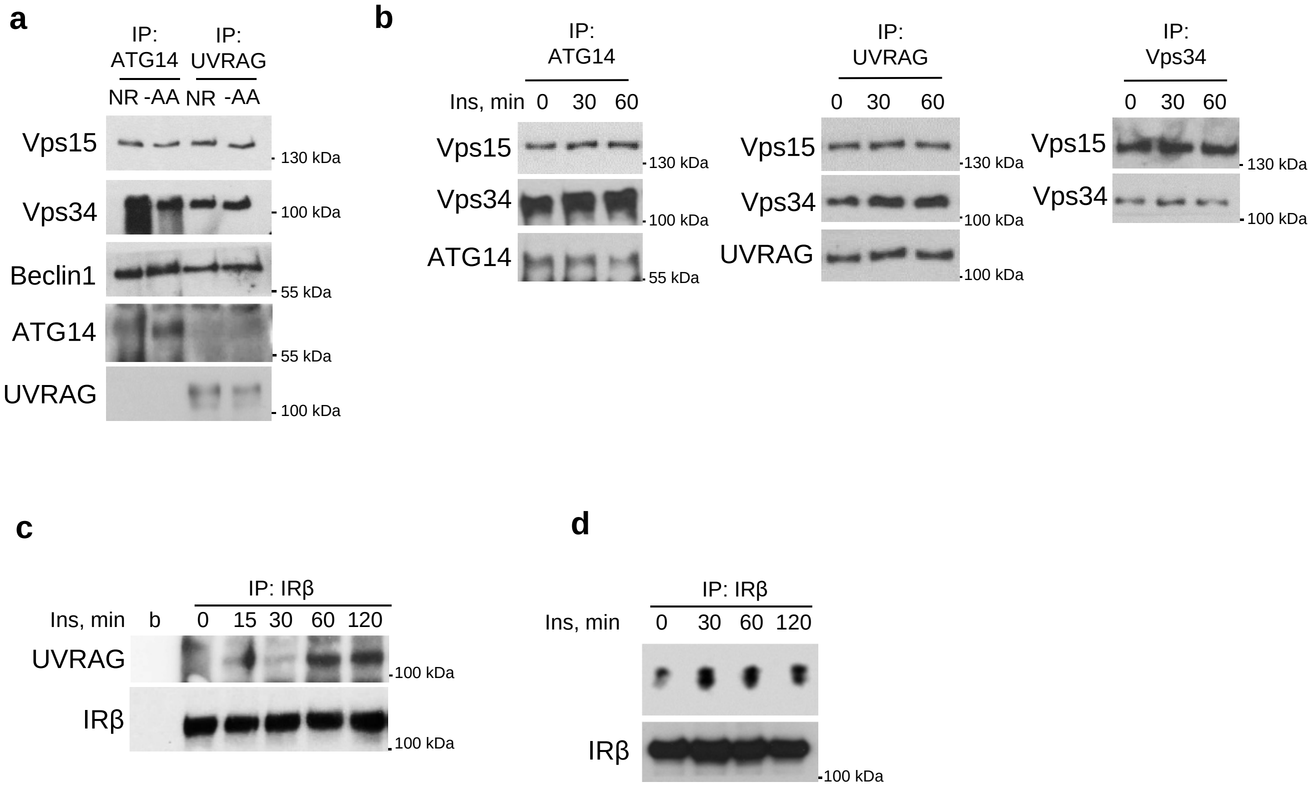

Supplementary Figure 3. Class III PI3K complexes upon insulin stimulation.
a. and **b.** Representative immunoblot analyses of immunoprecipitates used for the Vps34 *in vitro* kinase assays with indicated antibodies (presented in main Fig. 3a and Fig. 3b). **c.** Endogenous IRβ was immunoprecipitated from primary hepatocytes which were serum starved for 24 hours and stimulated with 1µM insulin for indicated times. The presence of UVRAG and IRβ in the immunoprecipitation eluates was revealed by immunoblot with indicated antibodies. The protein G beads served as a control of the non-specific binding. **d.** Class III PI3K activity was assayed in IRβ-immunoprecipitates from primary hepatocytes which were serum starved for 24 hours followed by stimulation with 1µM insulin for indicated times. Immunoblot with anti-IRb antibody presented below.

Supplementary Figure 4

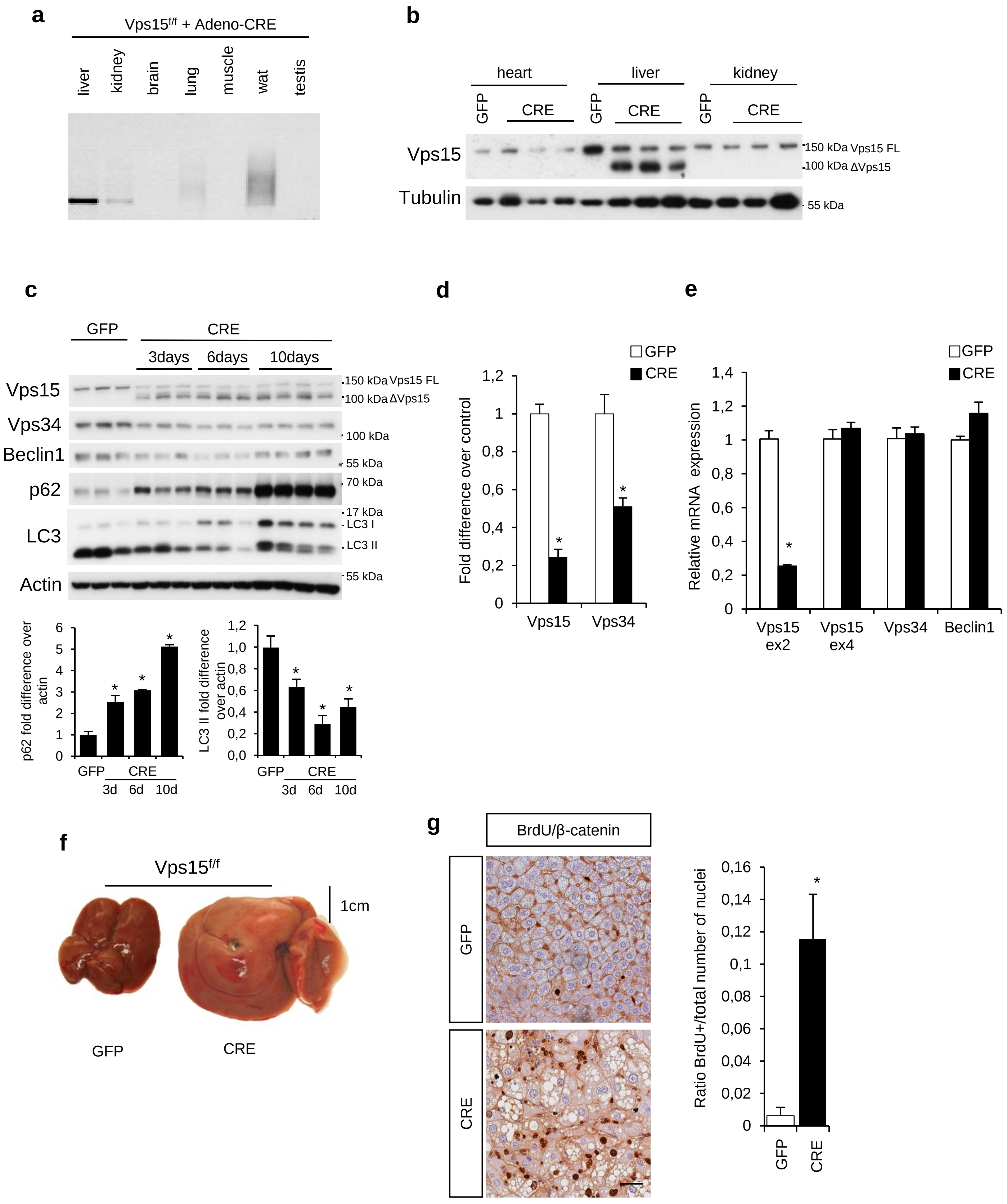

Supplementary Figure 4. Analyses of mice with acute hepatic *Vps15* depletion using Adeno-CRE vectors.

a. Representative PCR analysis to detect recombination of floxed *Vps15* allele after transduction with Adeno-Cre expressing vectors. Indicated tissues were dissected from $Vps15^{f/f}$ mice ten days post-transduction with Adeno-CRE vectors. Total DNA was extracted by alkaline method and the recombination region amplified by PCR with specific set of primers. **b.** Immunoblot analysis of Vps15 expression in heart, liver and kidneys in random-fed $Vps15^{f/f}$ mice ten days post-transduction with Adeno-GFP or Adeno-CRE vectors. Probing with anti-tubulin antibody served as loading control. **c.** Immunoblot analyses of liver extracts of random-fed $Vps15^{f/f}$ mice sacrificed at different times post-transduction with Adeno-GFP or Adeno-CRE vectors using indicated antibodies. Densitometric quantification of p62 and LC3 II protein levels over actinpresented below (n=3-4, P<0.05 *: vs Adeno-GFP, 2-tailed, unpaired Student's t test).**d.** Densitometric quantification of Vps15 and Vps34 protein levels over GAPDH shows the degree of Vps15 and Vps34 downregulation in livers of Adeno-CRE injected mice sacrificed ten days post-transduction (n=5-6, P<0.05 *: vs Adeno-GFP, 2-tailed, unpaired Student's t test). **e.** Relative mRNA expression levels of class III PI3K complex subunits in the livers random-fed mice ten days post-transduction with Adeno-GFP or Adeno-CRE vectors. Data are means ±SEM (n=5-8, P<0.05 *: vs Adeno-GFP, 2-tailed, unpaired Student's t test). **f.** Macroscopic view of livers of random-fed mice ten days post-transduction with Adeno-GFP or Adeno-CRE vectors. **g.** Liver sections of random-fed mice were immunostained with anti-BrdU and anti-β-catenin antibodies to label proliferating cells (BrdU incorporation) and to facilitate morphometric analysis by labelling plasma membrane (β-catenin) (left panel). The analysis of hepatocyte proliferation is presented as a ratio of BrdU+ nuclei to total number of nuclei ±SEM (n=5-6, P<0.05 *: vs Adeno-GFP, 2-tailed, unpaired Student's t test), (right panel). Scale bar: 50 µm.

Supplementary Figure 5

a

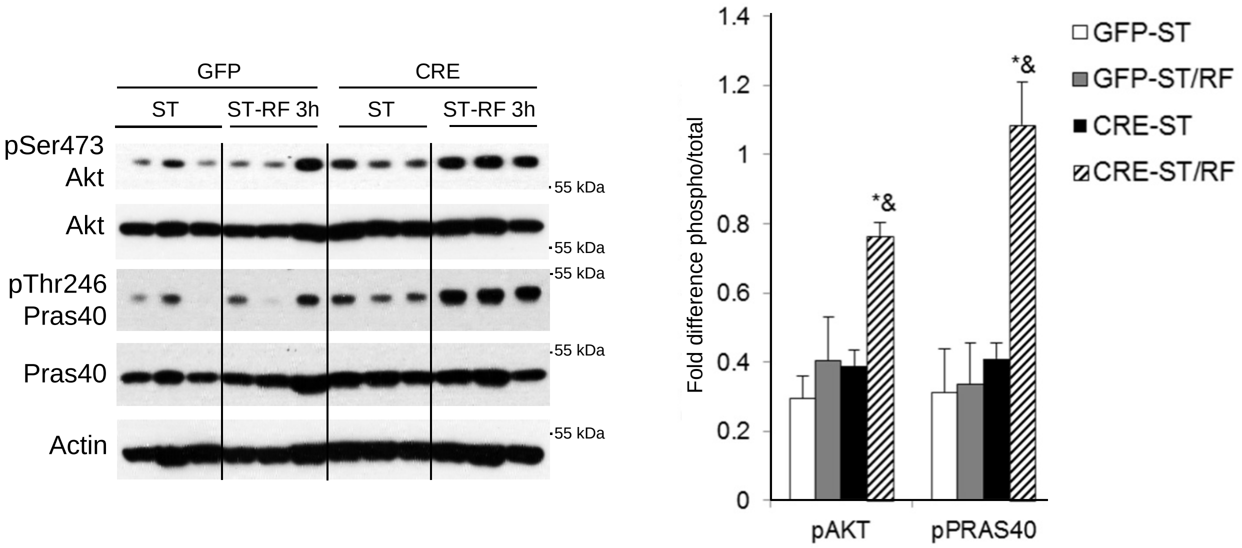

b

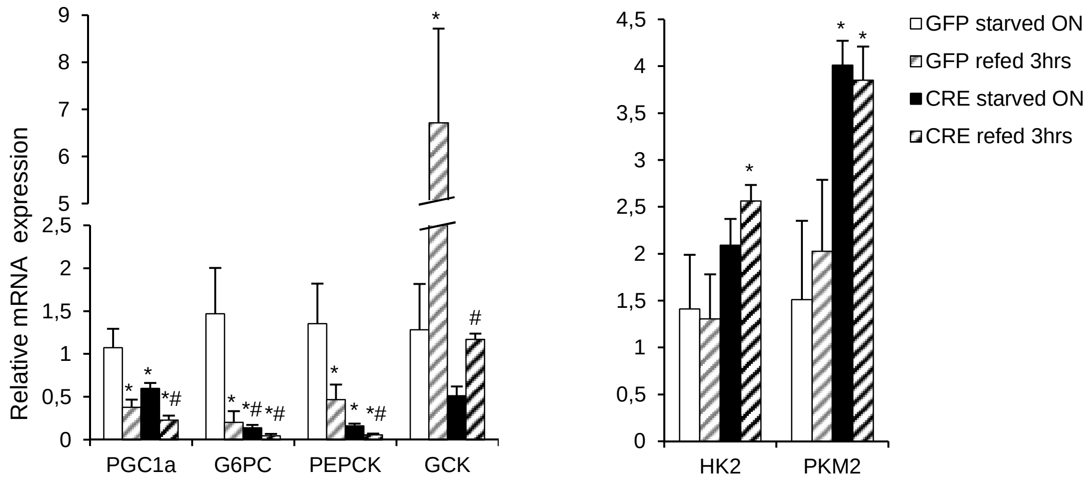

Supplementary Figure 5. Activated Akt responses in livers of mice with acute hepatic *Vps15* depletion using Adeno-CRE vectors.
a. Immunoblot analysis of hepatic Akt and Pras40 phosphorylation in response to three hours refeeding with standard chow. Vps15$^{f/f}$ mice nine days post-transduction were starved overnight followed by three hours refeeding before the sacrifice. Right panel presents densitometric analysis of normalised phospho protein to total protein levels in liver extracts of starved and refed mice. Data are means ±SEM (n=3, P<0.05 *: vs Adeno-GFP refed, &: vs Adeno-CRE starved, 2-tailed, unpaired Student's t test). **b.** Relative mRNA expression levels of genes implicated in gluconeogenesis and glycolysis in the livers of mice treated as in **a.** Data are means ±SEM (n=4-6, P<0.05 *: vs Adeno-GFP starved, #: vs Adeno-CRE starved, 2-tailed, unpaired Student's t test).

Supplementary Figure 6

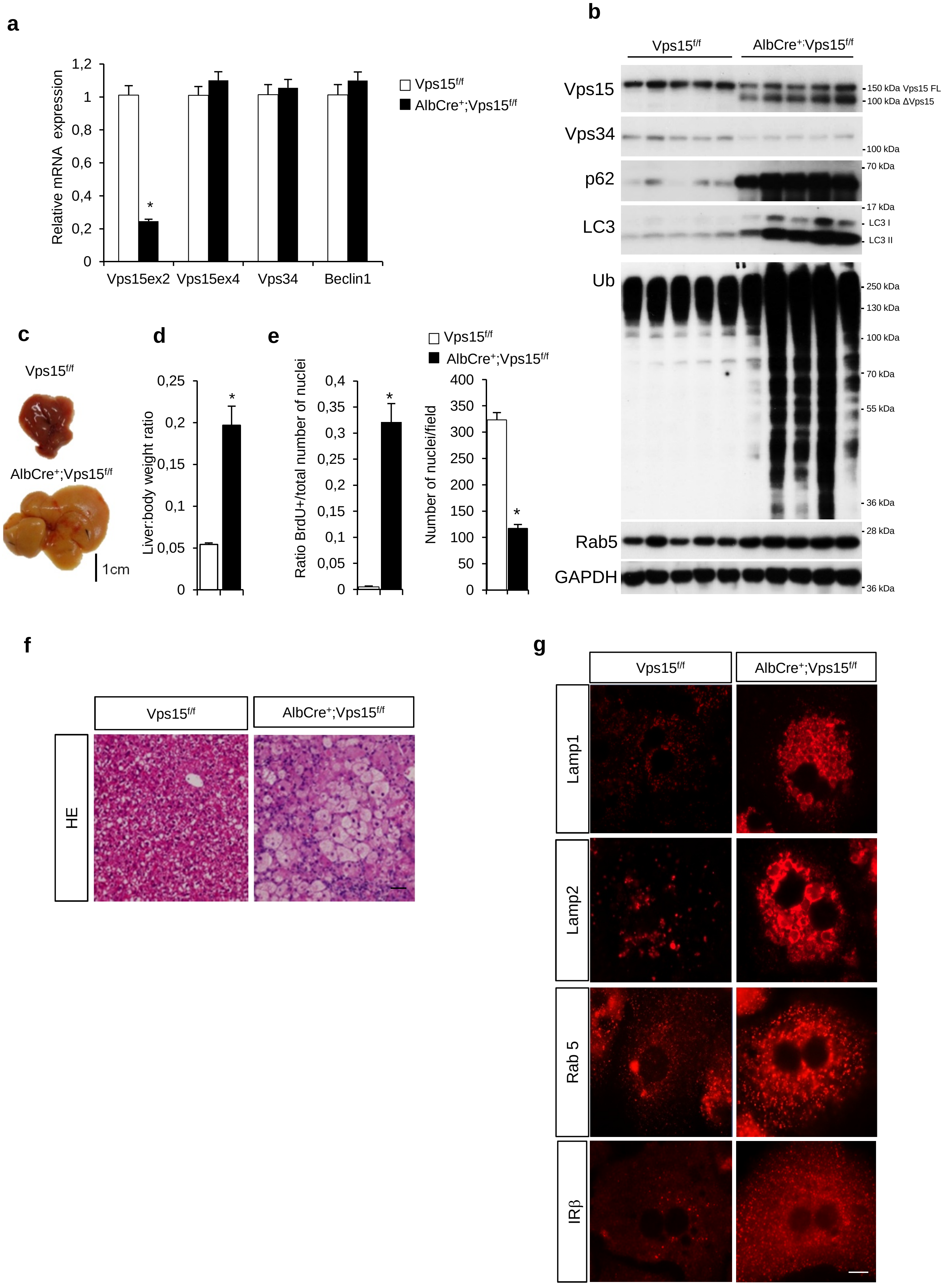

Supplementary Figure 6. Characterisation of hepatic *Vps15* mutants.

a. Relative mRNA expression levels of class III PI3K complex genes in the livers of one month old random-fed $Vps15^{f/f}$ and $AlbCre^{+}$;$Vps15^{f/f}$ mice. Data are means ±SEM (n=5-8, P<0.05 *: vs $Vps15^{f/f}$, 2-tailed, unpaired Student's t test). **b.** Immunoblot analysis of protein expression in livers of one month old random-fed $Vps15^{f/f}$ and $AlbCre^{+}$;$Vps15^{f/f}$ mice with indicated antibodies. Probing with anti-GAPDH antibody served as loading control. **c.** Macroscopic view of livers of one month old random-fed $Vps15^{f/f}$ and $AlbCre^{+}$;$Vps15^{f/f}$ mice. **d.** Liver weight to body weight ratio was determined in one month old random-fed $Vps15^{f/f}$ and $AlbCre^{+}$;$Vps15^{f/f}$ mice. Data are means ±SEM (n=8-11, P<0.05 *: vs $Vps15^{f/f}$, 2-tailed, unpaired Student's t test). **e.** Liver sections of random-fed mice were immunostained with anti-BrdU and anti-β-catenin antibodies to label proliferating cells (BrdU incorporation) and to facilitate morphometric analysis by labelling plasma membrane (β-catenin). The analysis of hepatocyte proliferation is presented as a ratio of BrdU+ nuclei to total number of nuclei ±SEM (n=3, P<0.05 *: vs $Vps15^{f/f}$, 2-tailed, unpaired Student's t test) (left panel). Increased size of Vps15-null hepatocytes is evident by the quantification of hepatocyte nuclei ±SEM (n=3, P<0.05 *: vs $Vps15^{f/f}$, 2-tailed, unpaired Student's t test) (right panel). **f.** H&E stained liver sections of one month old random-fed $Vps15^{f/f}$ and $AlbCre^{+}$;$Vps15^{f/f}$ mice. Scale bar: 50 µm. **g.** Microscopic analyses of subcellular localisation of endogenous IRβ, LAMP1, LAMP2 and Rab5 in primary hepatocytes isolated from livers of $Vps15^{f/f}$ and $AlbCre^{+}$;$Vps15^{f/f}$ mice revealed by indirect immunofluorescence using respective antibodies. Scale bar: 10 µm.

Supplementary Figure 7

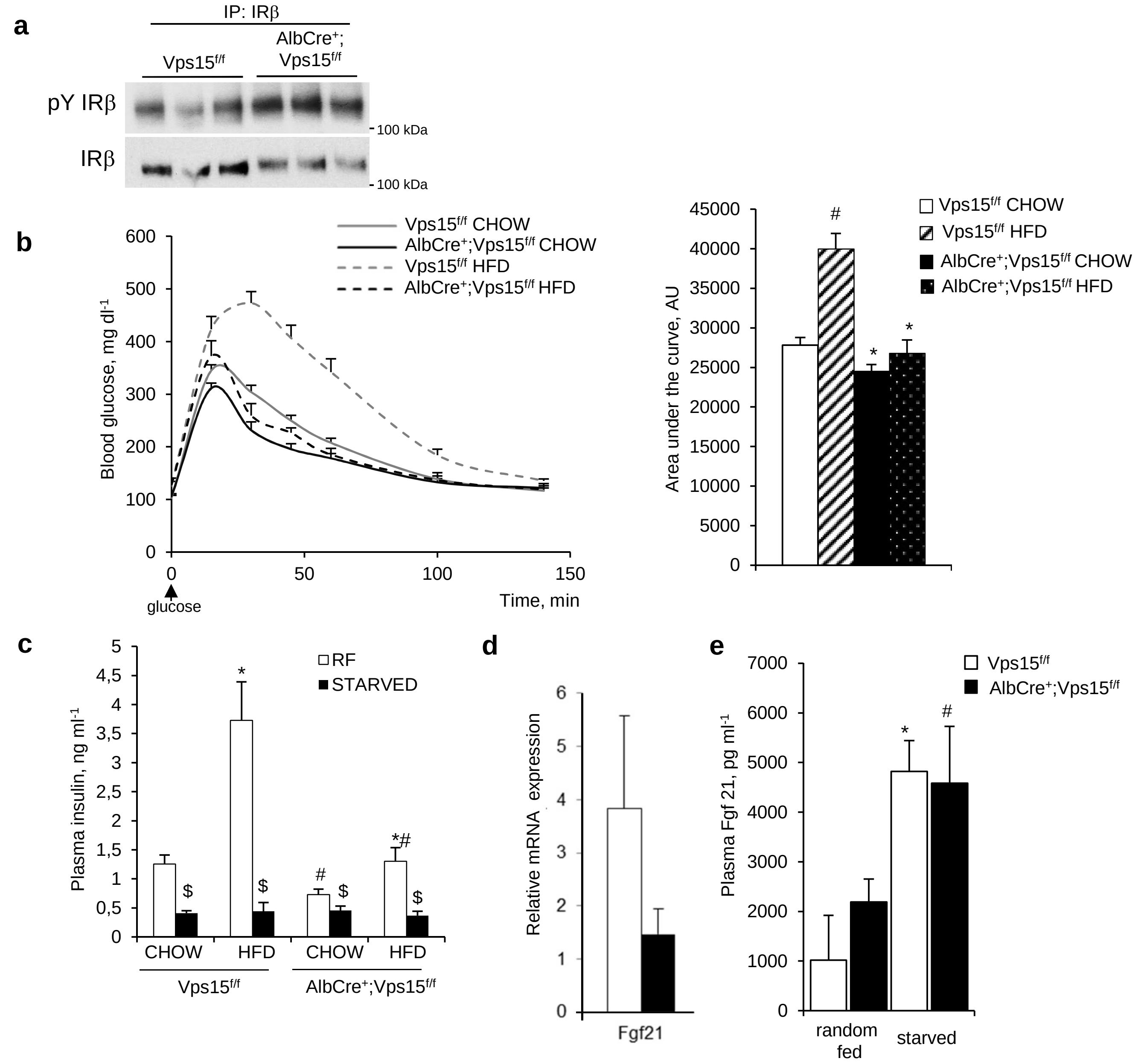

Supplementary Figure 7. Improved insulin signalling of hepatic Vps15 mutants.
a. Immunoblot analysis of IRb tyrosine phosphorylation in liver extracts of six week old Vps15$^{f/f}$ and AlbCre^{+};Vps15$^{f/f}$ mice. Mice were starved for six hours before intraperitoneal injection of 1u/kg of insulin. Livers were collected ten minutes after injection and phosphorylated at pY1146 IRb was detected in IRb immunoprecipitates using phospho-specific antibody. **b.** Intraperitoneal glucose tolerance test performed on overnight starved two month old Vps15$^{f/f}$ and AlbCre^{+};Vps15$^{f/f}$ mice subjected to HFD or CHOW regime for two weeks. The histogram of an average value of the area under the respective curve was calculated with GraphPadPrizm5 software. Data are means ±SEM (n=6-14, P<0.05 #: vs CHOW, *: vs Vps15$^{f/f}$, 2-tailed, unpaired Student's t test). **c.** Plasma insulin levels in six week old Vps15$^{f/f}$ and AlbCre^{+};Vps15$^{f/f}$ mice. Blood was collected retroorbitally in random fed or overnight starved mice. Data are means ±SEM (n=6-14, P<0.05 $: vs RF, *: vs CHOW, #: vs Vps15$^{f/f}$, 2-tailed, unpaired Student's t test). **d.** Relative mRNA expression levels of Fgf21 in livers of one month old random-fed Vps15$^{f/f}$ and AlbCre^{+};Vps15$^{f/f}$ mice. Data are means ±SEM (n=5-8, P<0.05 *: vs Vps15$^{f/f}$, 2-tailed, unpaired Student's t test). **e.** Plasma concentration of Fgf21 in six week old random-fed and overnight starved (16 hours) Vps15$^{f/f}$ and AlbCre^{+};Vps15$^{f/f}$ mice measured by ELISA kit from R&D Systems as recommended by manufacturer. Data are means ±SEM (n=6-9, P<0.05 *: vs Vps15$^{f/f}$ random fed, #: vs AlbCre^{+};Vps15$^{f/f}$ random fed, 2-tailed, unpaired Student's t test).

Supplementary Figure 8

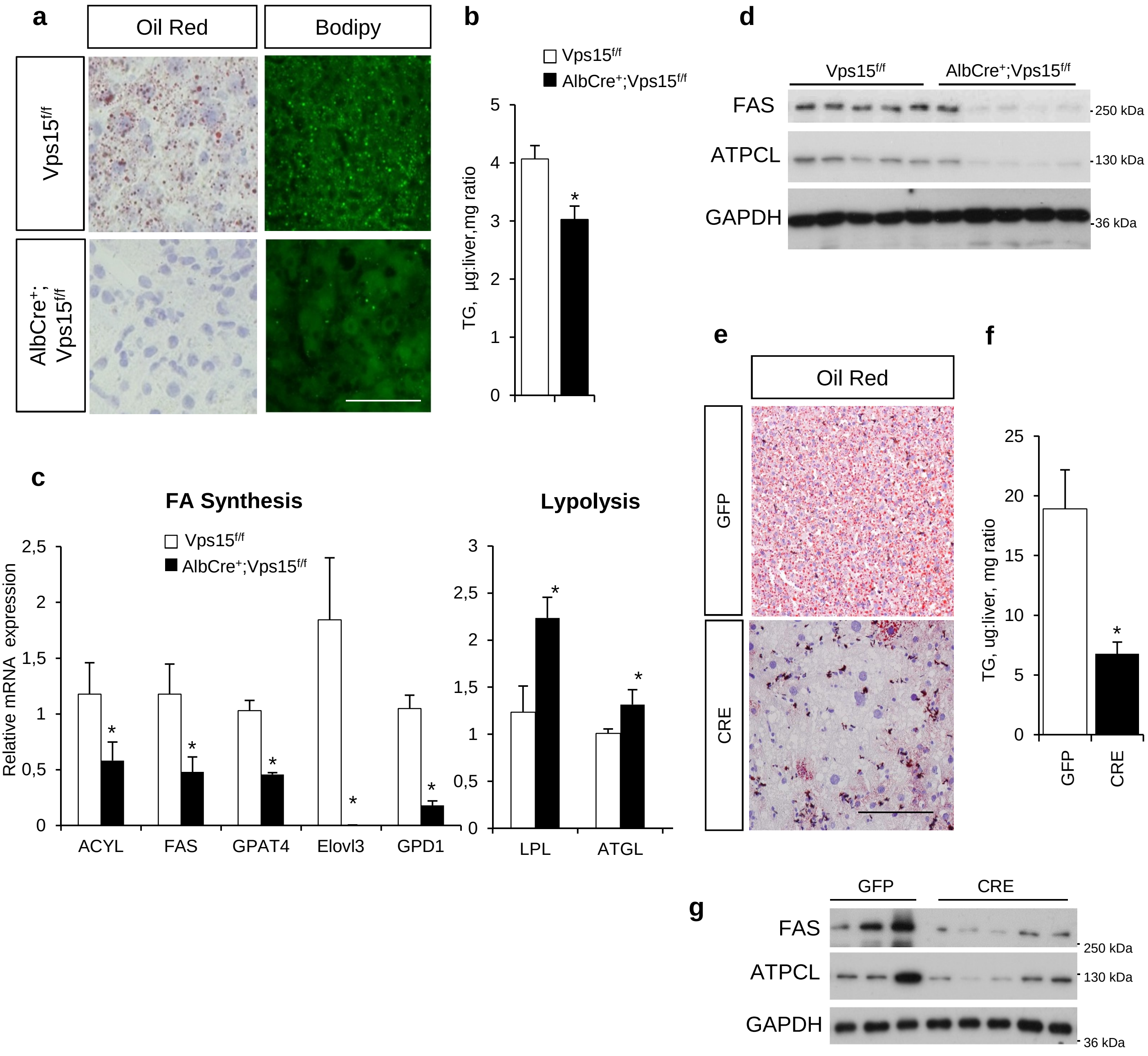

Supplementary Figure 8. Lipid metabolism in hepatic *Vps15* mutants.
a., **b.**, **c.** and **d.** Microscopic analyses of frozen liver tissue sections by Oil Red and Bodipy staining (**a**), hepatic triglyceride concentration (**b**), relative mRNA expression levels of the genes involved in the lipogenesis (**c**) and immunoblot analysis of lipogenic enzymes expression (**d**) in the livers of one month old random-fed Vps15$^{f/f}$ and AlbCre^{+};Vps15$^{f/f}$ mice. Data are means ±SEM (n=5-8, P<0.05 *: vs Vps15f/f, 2-tailed, unpaired Student's t test). Scale bar: 100 µm.
Oil Red staining of frozen liver tissue sections. Scale bar: 100 µm. (**e**), hepatic triglyceride concentration (**f**) and immunoblot analysis of lipogenic enzymes expression in livers of random-fed mice ten days post-transduction with Adeno-GFP or Adeno-CRE vectors (**g**). Data are means ±SEM (n=5-6, P<0.05 *: vs Adeno-GFP, 2-tailed, unpaired Student's t test).

Supplementary Figure 9

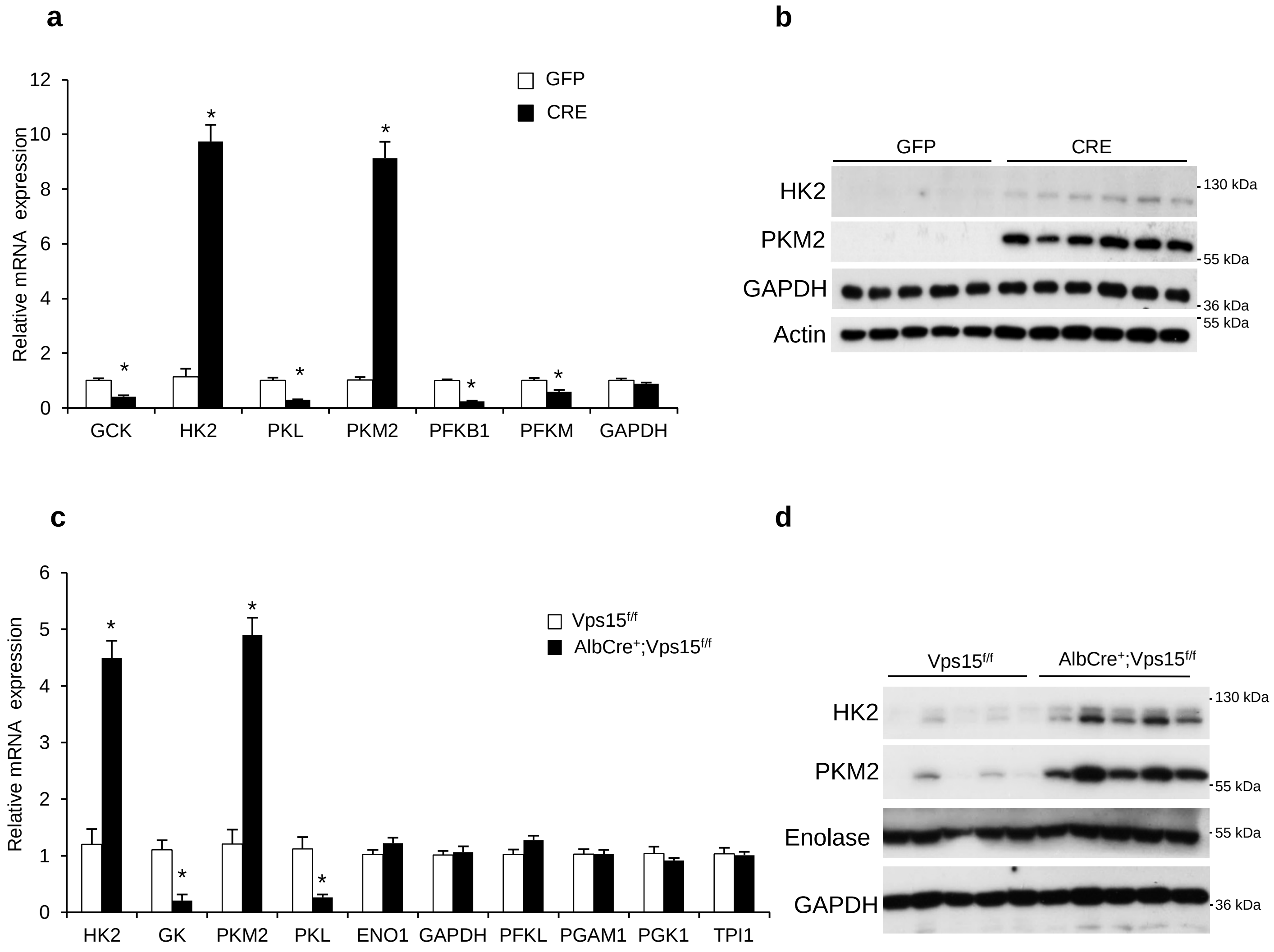

Supplementary Figure 9. Upregulation of glycolytic gene expression in hepatic Vps15 mutants. Relative mRNA expression levels (**a**) and protein levels (**b**) of the glycolytic enzymes in the livers of one month old random-fed Vps15$^{f/f}$ and AlbCre^{+};Vps15$^{f/f}$ mice. Immunoblot with anti-actin antibody served as loading control. Data are means ±SEM (n=5-8, P<0.05 *: vs Vps15$^{f/f}$, 2-tailed, unpaired Student's t test). Relative mRNA expression levels (**c**) and protein levels (**d**) of the glycolytic enzymes in livers of random-fed mice ten days post-transduction with Adeno-GFP or Adeno-CRE vectors. Immunoblot with anti-GAPDH antibody served as loading control. Data are means ±SEM (n=5-6, P<0.05 *: vs Adeno-GFP, 2-tailed, unpaired Student's t test).

Supplementary Figure 10

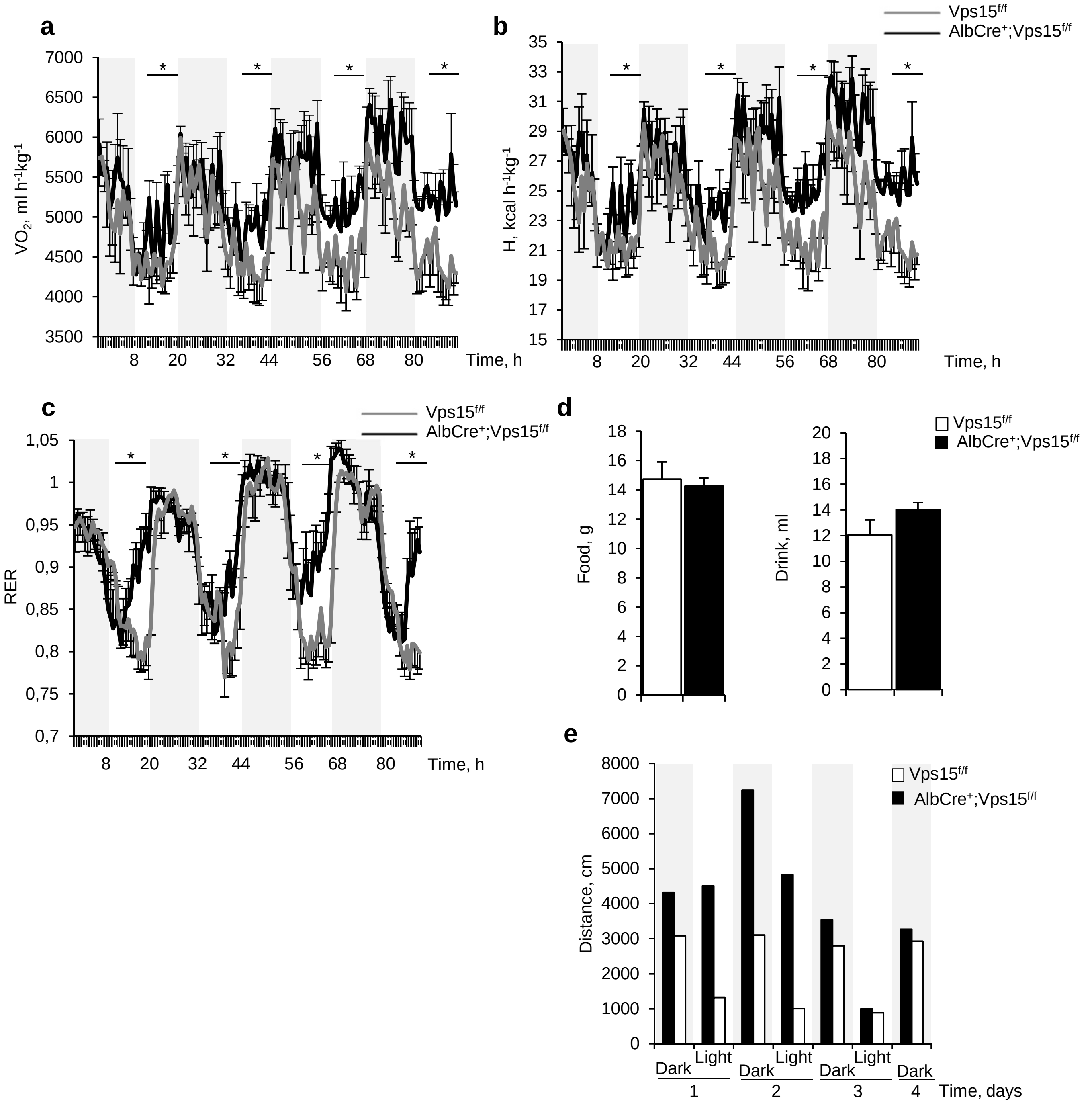

Supplementary Figure 10. *In vivo* metabolic profiling of hepatic Vps15 mutants. Real time O2 consumption (VO2) (**a**), energy expenditure (EE) (**b**), RER (**c**), cumulative food and drink consumption (**d**) and cumulative distance over the 12 hour period (**e**) was measured in six week old Vps15$^{f/f}$ and AlbCre^{+};Vps15$^{f/f}$ littermates. Mice were placed individually into an eight cage open circuit system equipped with an air pump, a control unit, a sample switch unit to draw air samples from the cages, and an air drying unit (LabMaster system; TSE System). Prior to recording mice were adapted to new environment for 48 hours. The food and water intake, physical activity of every ambulatory movement, O2 uptake and CO2 production were measured continuously at 9-minute intervals. The RER values were calculated from the O2 consumption (VO2) (**a**) and CO2 production (VCO2) relative to body weight. Use of infrared sensors for detection of movement allowed continuous recording in both light and dark phases. Data collected from the last three days of housing were used to calculate all parameters for which results are reported. Measurements were extracted for every 45 min. Grey stripes on *x* axis indicate lights-off. Data are means ±SEM (n=4, P<0.05 *: vs Vps15f/f, 2-tailed, unpaired Student's t test).

Supplementary Figure 11

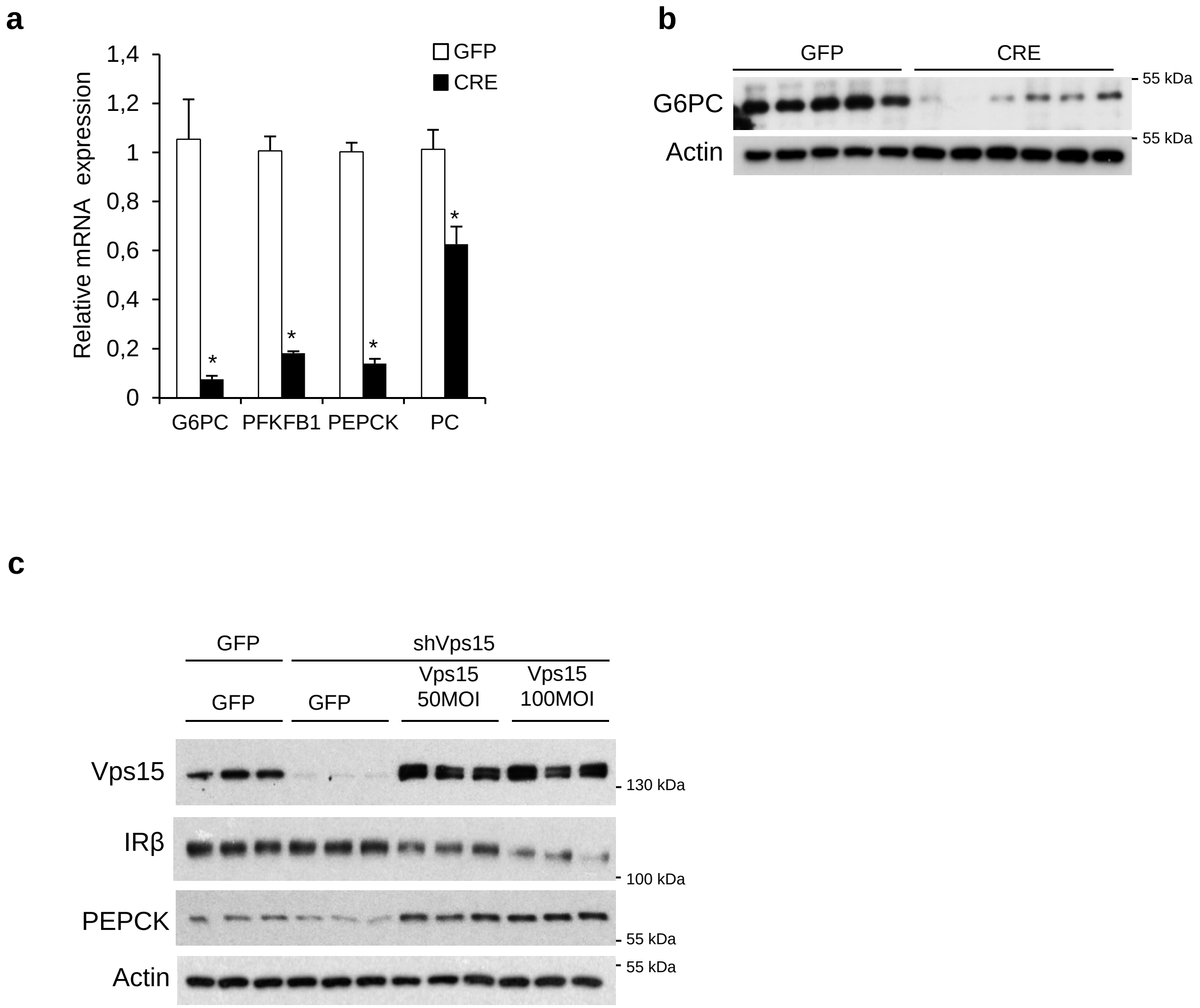

Supplementary Figure 11. Hepatic expression of the enzymes implicated in gluconeogenesis is blunted upon acute Vps15 depletion.

a. Relative mRNA expression levels of genes implicated in the gluconeogenesis in the livers random-fed mice ten days post-transduction with Adeno-GFP or Adeno-CRE vectors. Data are means ±SEM (n=5-6, P<0.05 *: vs Adeno-GFP, 2-tailed, unpaired Student's t test). **b.** Immunoblot analysis of total protein liver extracts of random-fed mice ten days post-transduction with Adeno-GFP or Adeno-CRE vectors using G6PC antibody. Probing with anti-actin antibody served as loading control. **c.** Immunoblot analyses of IRb and PEPCK protein levels in primary hepatocytes transduced with Adeno-GFP, Adeno-shRNA Vps15 or Adeno-shRNA Vps15 in combination with Adeno-hVps15 cDNA expressing adenoviruses. Cells were collected 48 hours post-infection. Immunoblot with anti-actin antibody served as a loading control.

Supplementary Figure 12

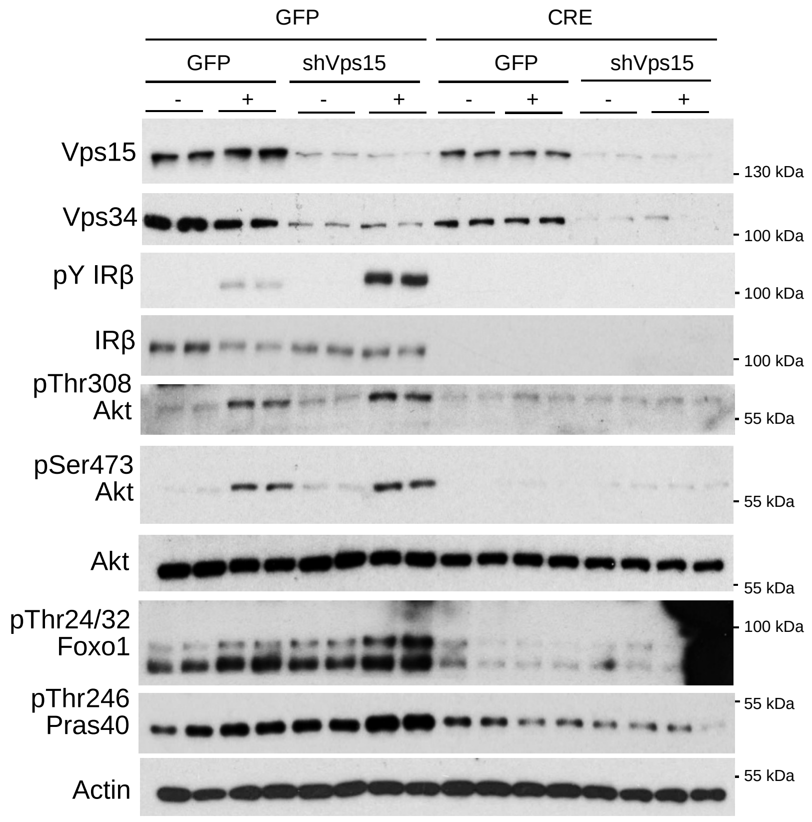

Supplementary Figure 12. Intact IR is required for Akt activation in cells upon acute Vps15 depletion.

Primary hepatocytes prepared from IR$^{f/f}$ mutant mice were transduced with Adeno-GFP or Adeno-CRE adenoviral vectors in combination with Adeno-GFP or Adeno-shRNA Vps15 expressing vectors. 24 hours post-transduction cells were starved and then stimulated with 1µM insulin for 1 hour. Activation of IR-signalling pathway was revealed by immunoblot analysis with indicated antibodies. Immunoblot with anti-actin antibody served as a loading control.

Supplementary Figure 13

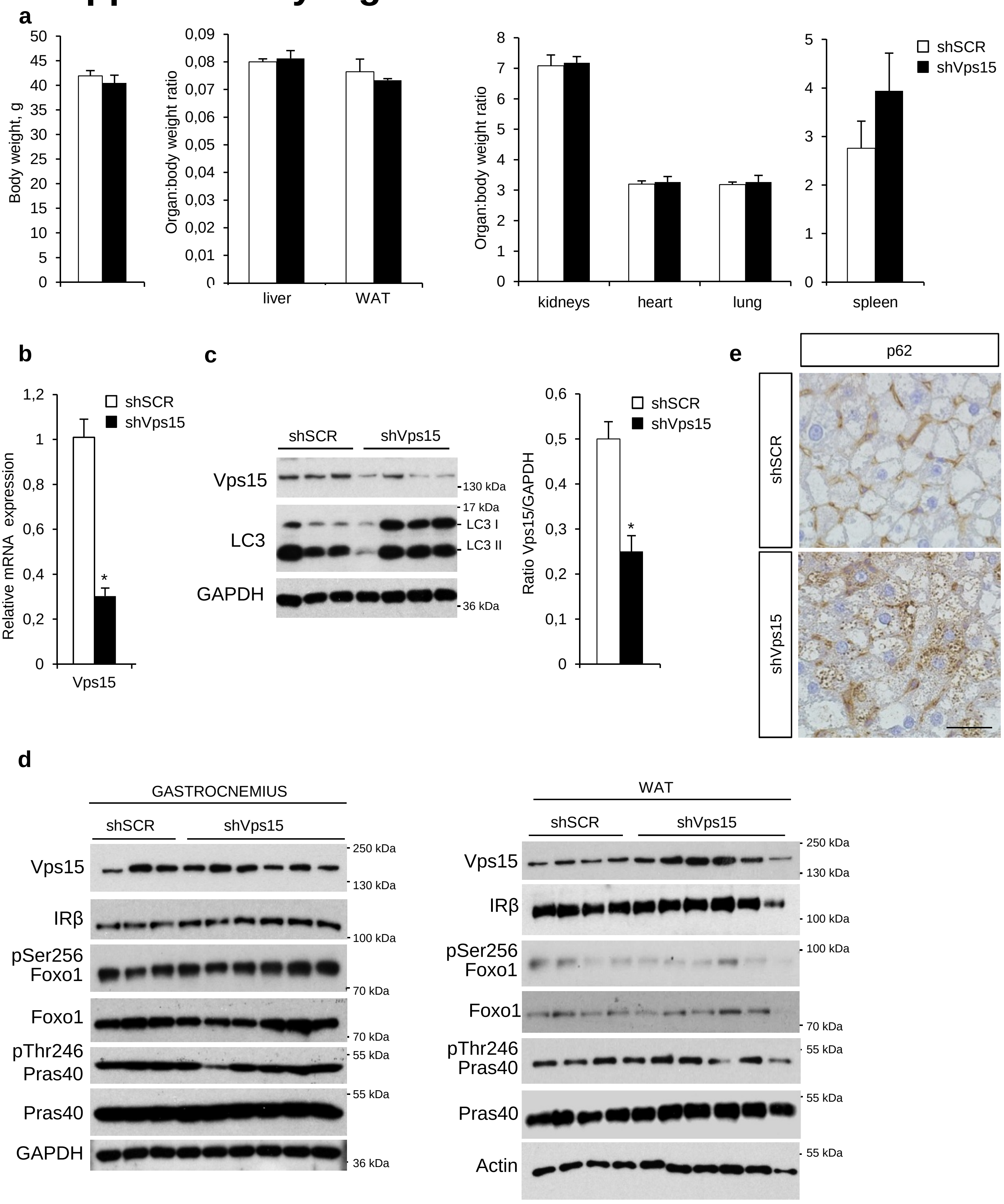

Supplementary Figure 13. Metabolic consequences of hepatic Vps15 downregulation observed in *ob/ob* and HFD-fed wild type mice.

a. Body weight and ratio of different organs to body weight in random-fed *ob/ob* mice eight days post-transduction with Adeno-shRNA SCR or Adeno-shRNA Vps15 vectors. Data are means ±SEM (n=4-6). **b.** Relative mRNA expression levels of Vps15 in the livers of random-fed *ob/ob* mice eight days post-transduction with Adeno-shRNA SCR or Adeno-shRNA Vps15 vectors. Data are means ±SEM (n=4-6, $P<0.05$ *: vs Adeno-shRNA SCR, 2-tailed, unpaired Student's t test). **c.** Immunoblot analysis of liver extracts of random-fed *ob/ob* mice eight days post-transduction with Adeno-shRNA SCR or Adeno-shRNA Vps15 vectors with indicated antibodies (left panel). Densitometric analysis of Vps15 levels relative to GAPDH in livers of mice injected with adenoviral vectors. Data are means ±SEM (n=4-6, $P<0.05$ *: vs Adeno-shRNA SCR, 2-tailed, unpaired Student's t test) (right panel). **d.** Immunoblot analysis of total protein extracts from gastrocnemius muscle and epididymal adipose tissue (WAT) of random-fed *ob/ob* mice eight days post-transduction with Adeno-shRNA SCR or Adeno-shRNA Vps15 vectors. **e.** Immunohistochemical staining using anti-p62 antibody of formol-fixed paraffin embedded liver sections of random-fed *ob/ob* mice eight days post-transduction with Adeno-shRNA Scrambled or Adeno-shRNA Vps15 vectors. Scale bar: 50 µm.

Supplementary Figure 14

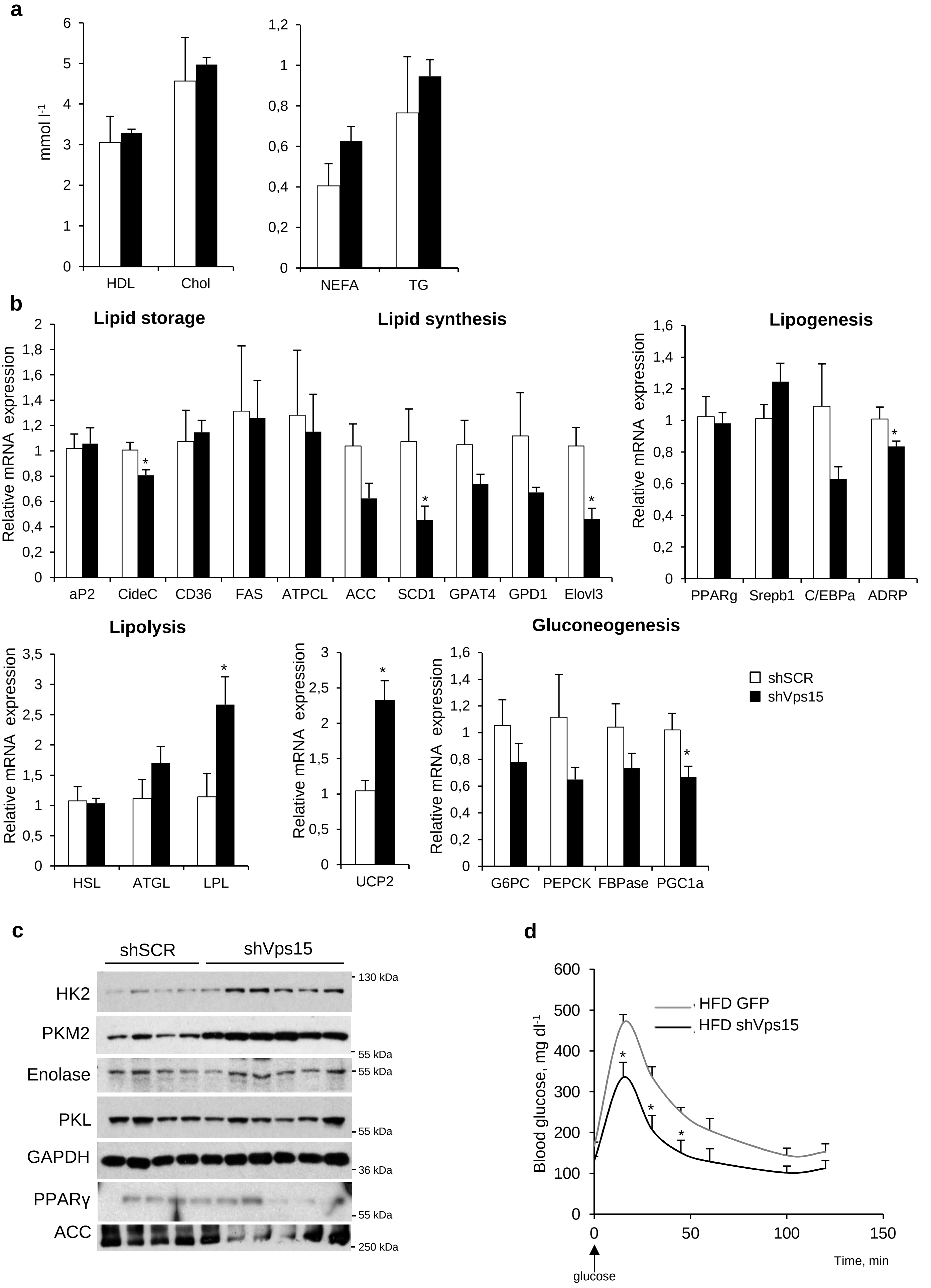

Supplementary Figure 14. Expression profiling of metabolic enzyme expression in livers of *ob/ob* mice transduced with Adeno-shRNA Vps15 vectors.

a. Metabolite analyses in plasma of random-fed *ob/ob* mice 8 days post-transduction with Adeno-shRNA SCR or Adeno-shRNA Vps15 vectors. Data are means ±SEM (n=4-6). **b.** Relative mRNA expression levels of genes implicated in the indicated metabolic pathways in the livers of random-fed *ob/ob* mice 8 days post-transduction with Adeno-shRNA SCR or Adeno-shRNA Vps15 vectors. Data are means ±SEM (n=4-6, P<0.05 *: vs Adeno-shRNA SCR, 2-tailed, unpaired Student's t test). **c.** Immunoblot analysis of liver extracts of random-fed *ob/ob* mice 8 days post-transduction with Adeno-shRNA SCR or Adeno-shRNA Vps15 vectors with indicated antibodies. **d.** Intraperitoneal glucose tolerance test of overnight starved wild type mice 7 days post-transduction with Adeno-GFP or Adeno-shRNA Vps15 vectors. Prior to injection with adenoviral vectors mice were submitted to HFD regime during 2 weeks. Data are means ±SEM (n=4 P<0.05 *: vs Adeno-GFP, 2-tailed, unpaired Student's t test).

Supplementary Figure 15

Figure 1a

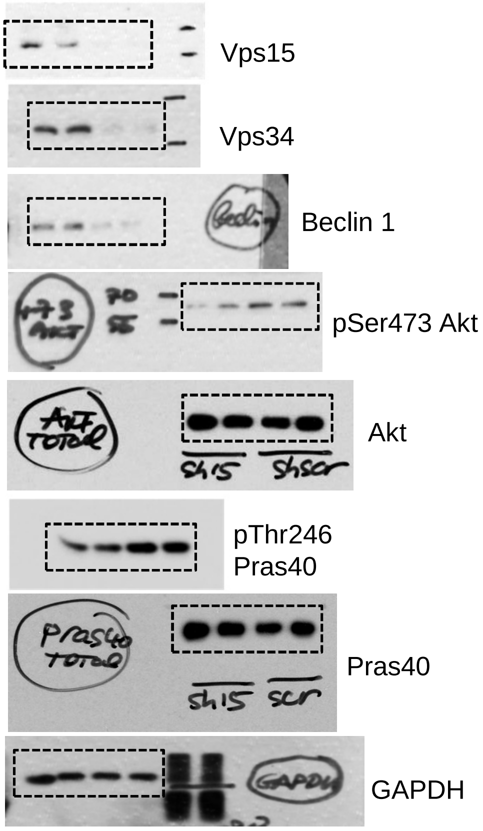

Figure 1b

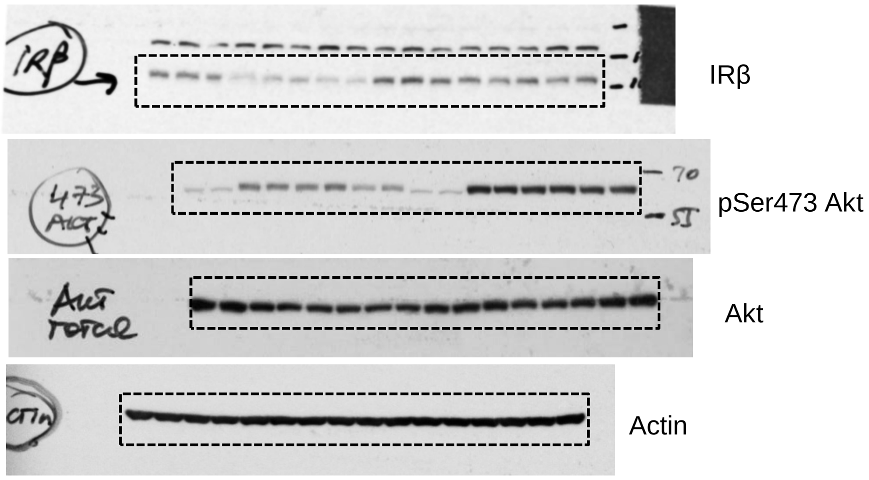

Figure 1c

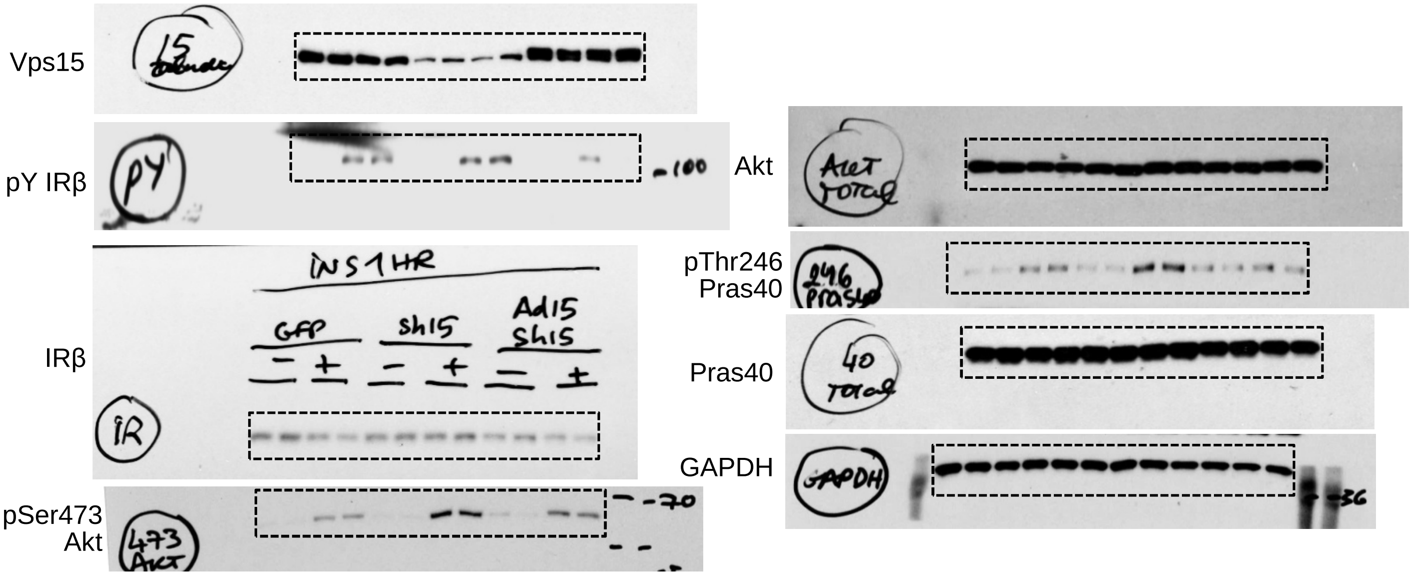

Supplementary Figure 15. Full scans of uncropped immunoblots presented in the main figures. Dashed boxes indicate the cropped regions.

Figure 1e

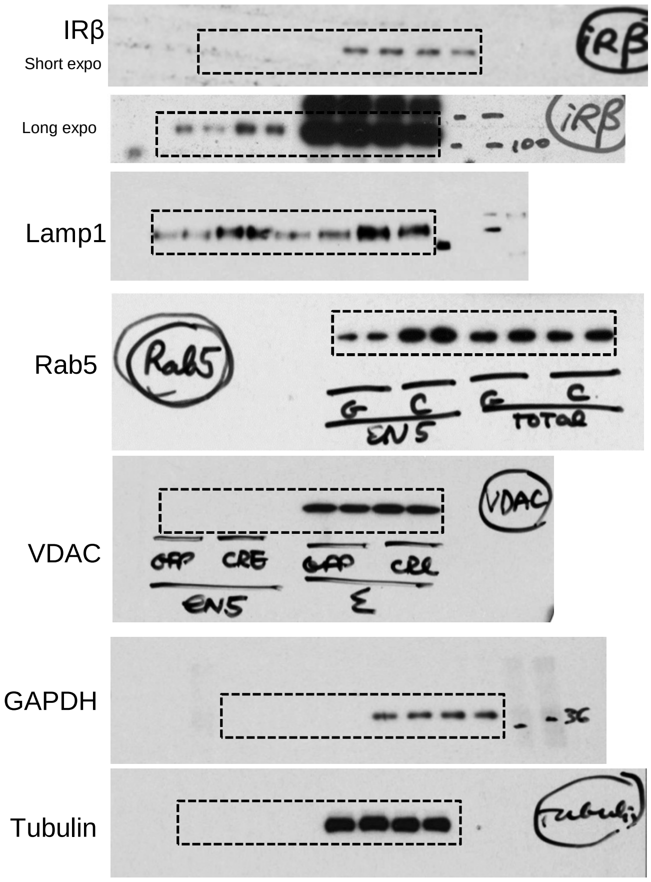

Figure 2d

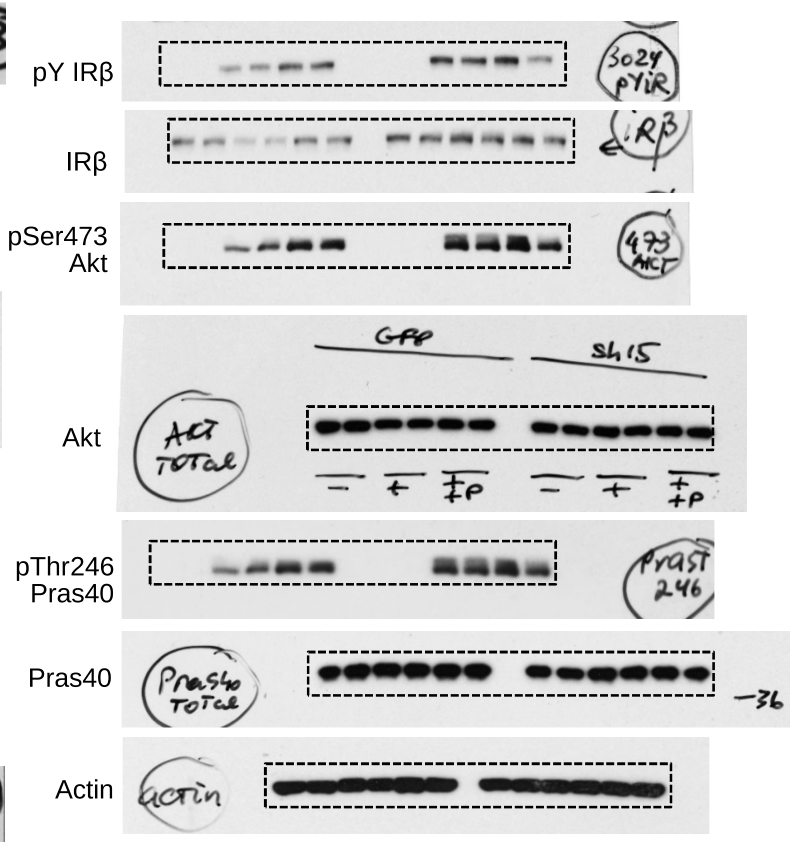

Figure 3c

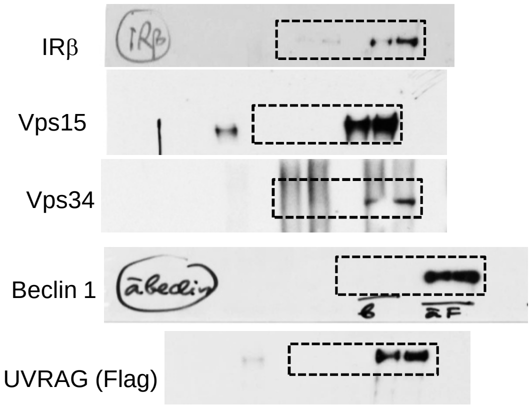

Figure 3d

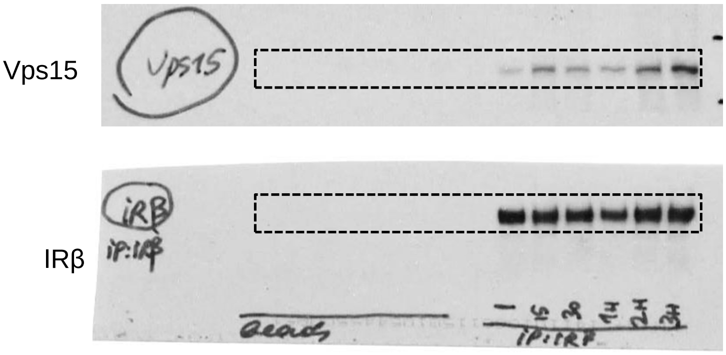

Figure 3e

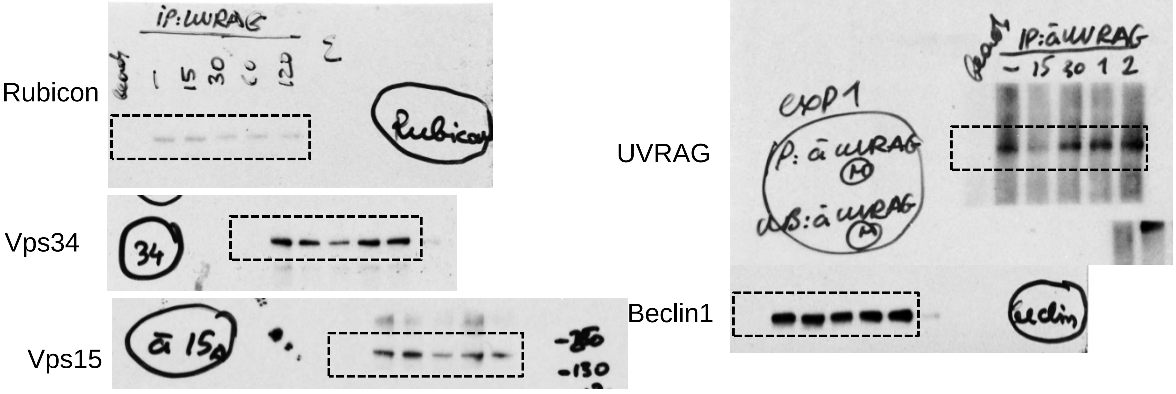

Supplementary Figure 15 - continued

Figure 4d

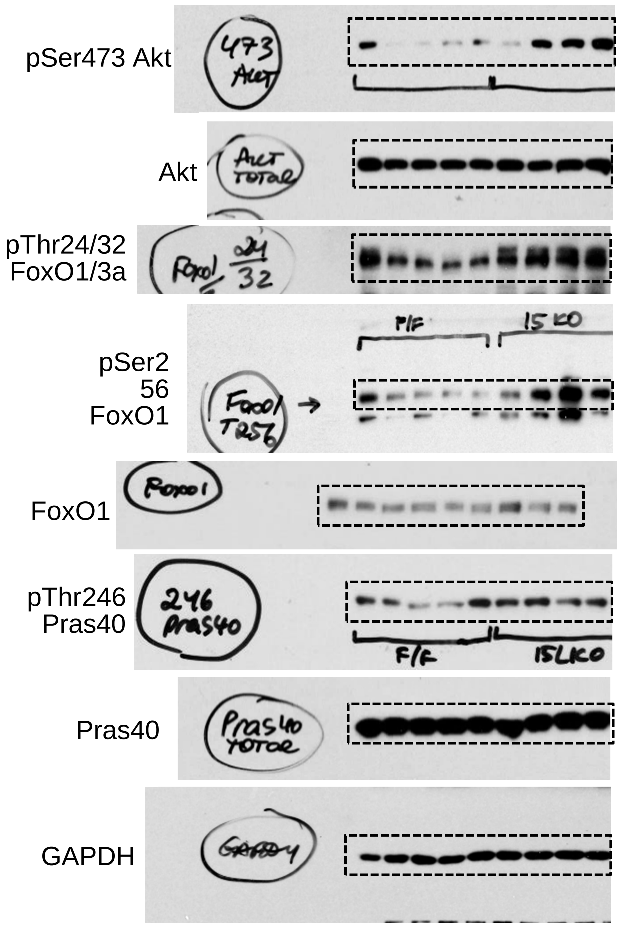

Figure 4e

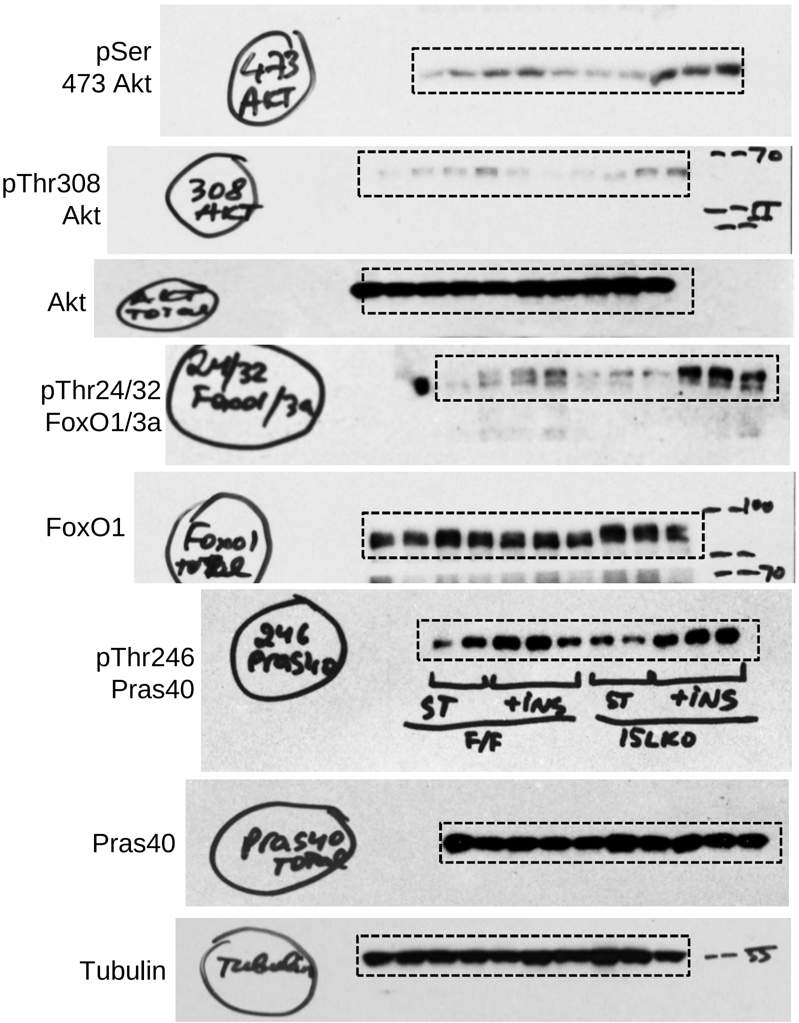

Figure 5b

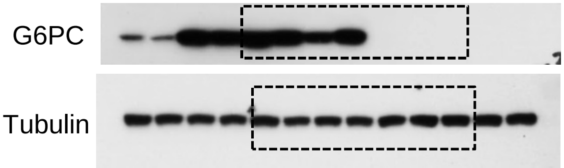

Figure 5d

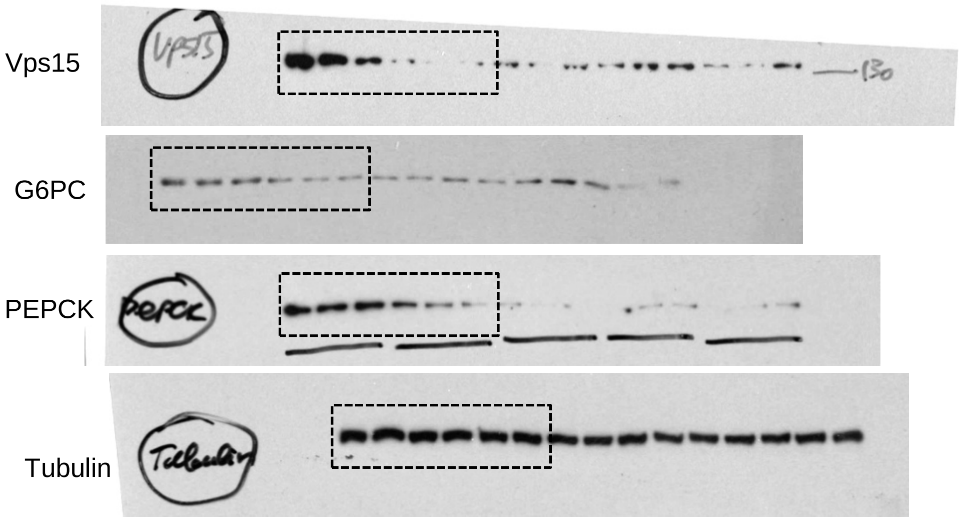

Supplementary Figure 15 - continued

Figure 6a

FoxO1

GST

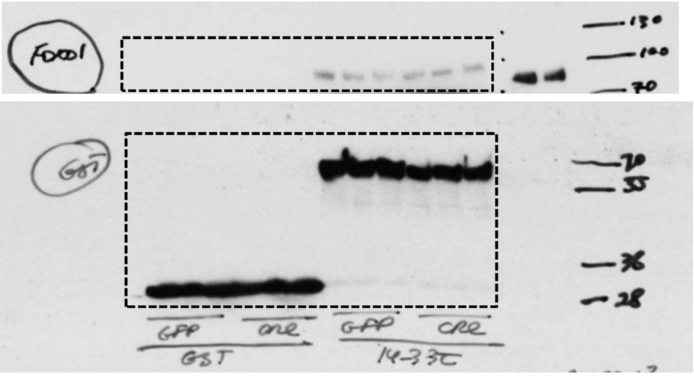

FoxO1

Actin

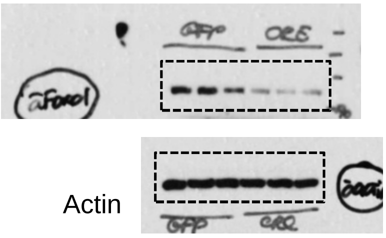

Figure 6b

FoxO1

Actin

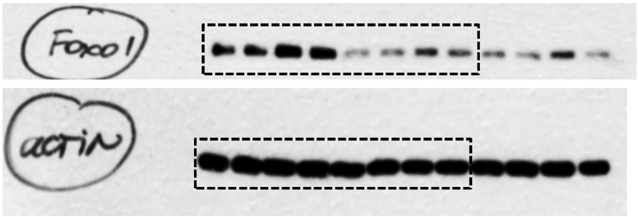

Figure 6c

FoxO1

GAPDH

Lamin A/C

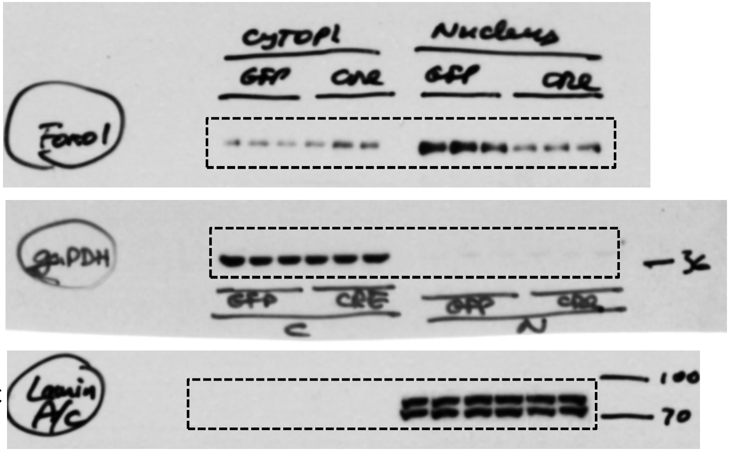

Supplementary Figure 15 - continued

Figure 7c

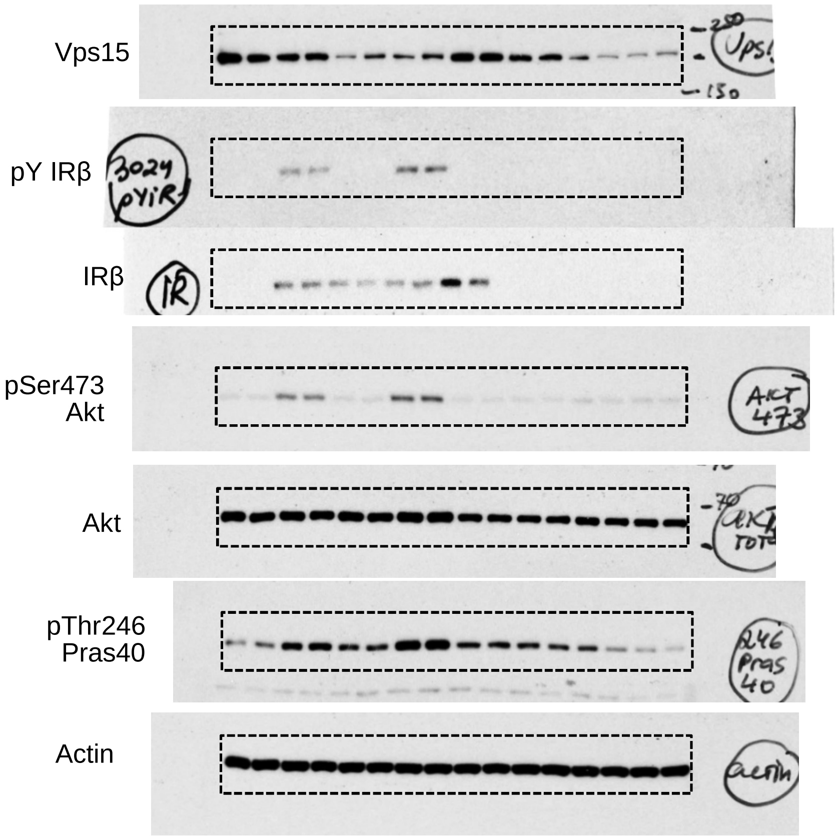

Figure 7d

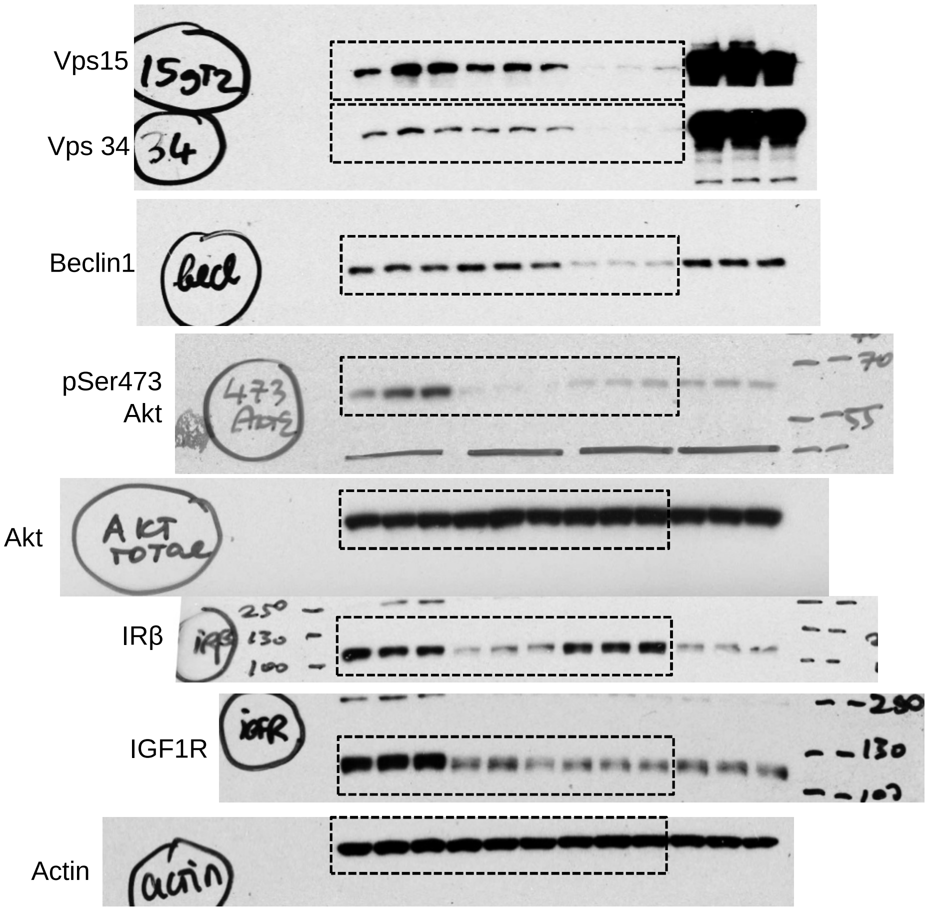

Supplementary Figure 15 - continued

Figure 7e

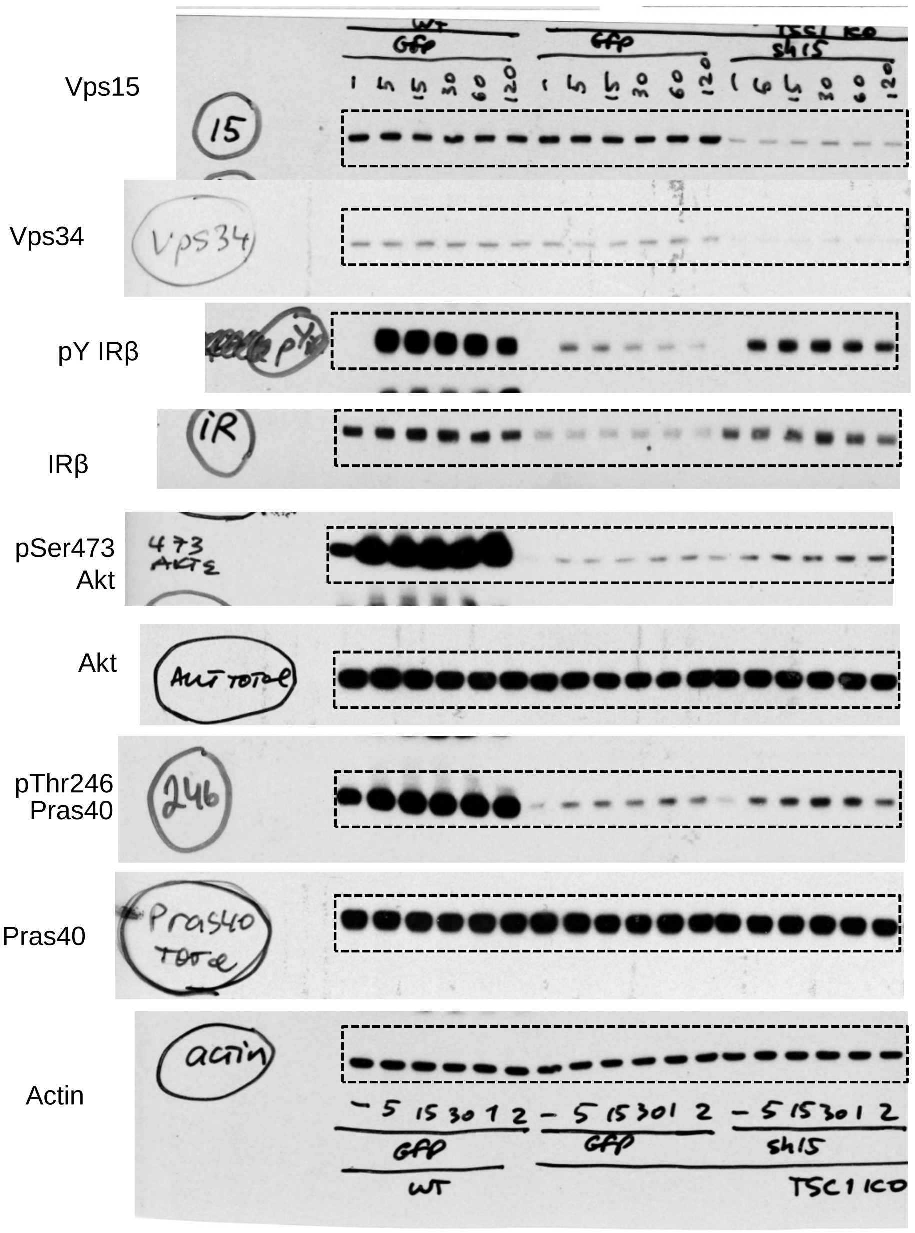

Supplementary Figure 15 - continued

Figure 8d

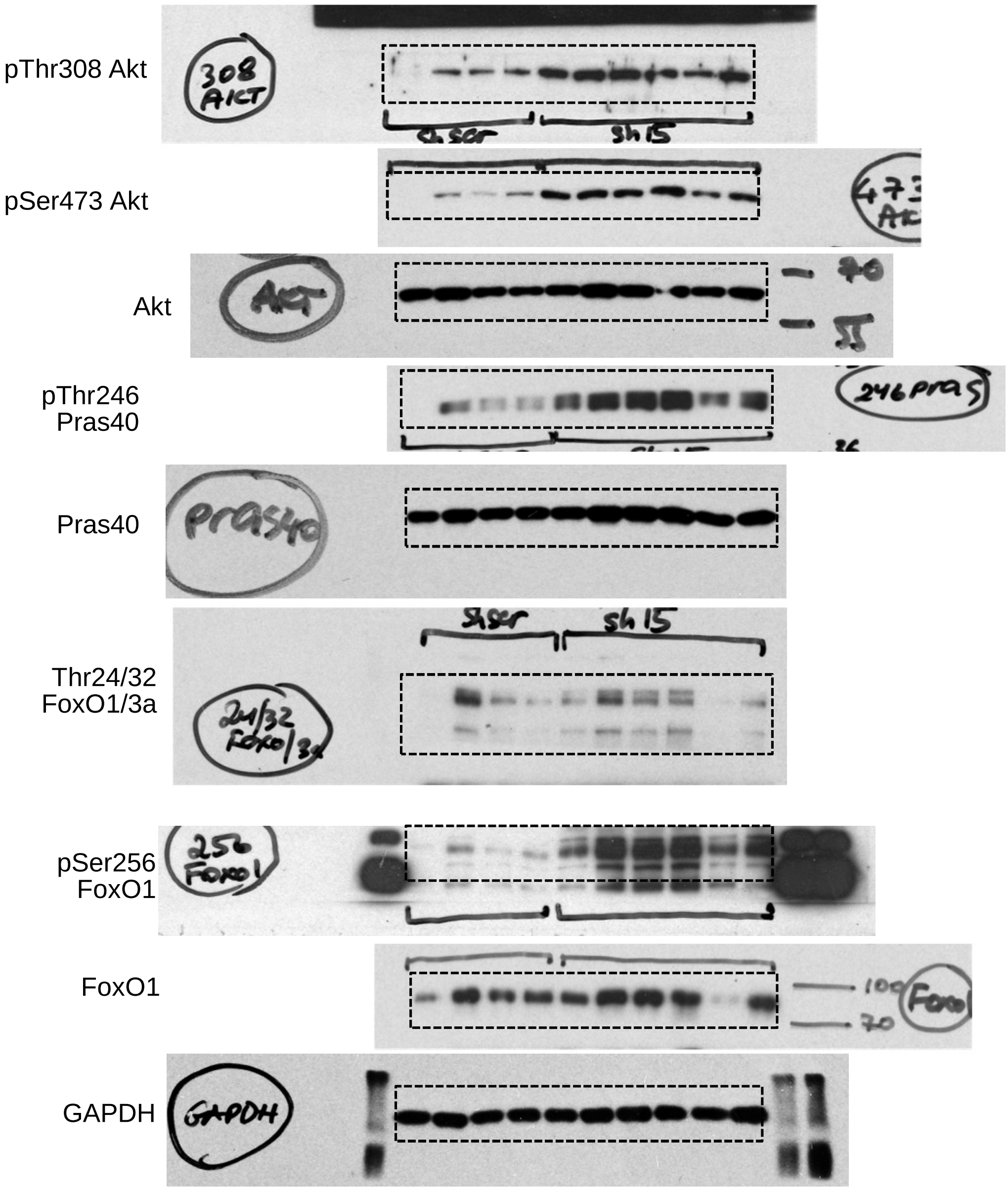

Figure 8e

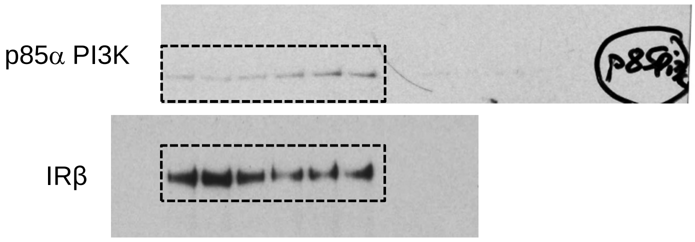

Supplementary Figure 15 - continued

Supplementary Table 1
Primer sequences used for RT-QPCR analysis and genotyping

Vps15 ex4	sense	5'-TTGTCCTGGTGTCCGTGATA-3'
	antisense	5'-GAGTGTCCTCAGGGCTTCAG-3'
Vps15 ex2	sense	5'-CCTGGTGGTTGTGAAGGTCT-3'
	antisense	5'-AGCGCTTCTCGATGTTGTTT-3'
Vps34	sense	5'-CCTGGACATCAACGTGCAG-3'
	antisense	5'-TGTCTCTTGGTATAGCCCAGAAA-3'
Beclin1	sense	5'-TGAATGAGGATGACAGTGAGCA-3'
	antisense	5'-CACCTGGTTCTCCACACTCTTG-3'
PGC1a	sense	5'-GGAATGCACCGTAAATCTGC-3'
	antisense	5'-TTCTCAAGAGCAGCGAAAGC-3'
G6PC	sense	5'-ATGAACATTCTCCATGACTTTGGG-3'
	antisense	5'-GACAGGGAACTGCTTTATTATAGG-3'
PEPCK	sense	5'-CTGCATAACGGTCTGGACTTC-3'
	antisense	5'-CAGCAACTGCCCGTACTCC-3'
GCK	sense	5'-TGAGCCGGATGCAGAAGGA-3'
	antisense	5'-CTCCCAGGTCTAAGGAGAGAAA-3'
PKM2	sense	5'-TCGCATGCAGCACCTGATT-3'
	antisense	5'-CCTCGAATAGCTGCAAGTGGTA-3'
HK2	sense	5'-TGATCGCCTGCTTATTCACGG-3'
	antisense	5'-AACCGCCTAGAAATCTCCAGA-3'
PPARγ	sense	5'-TGTGGGGATAAAGCATCAGGC-3'
	antisense	5'-CCGGCAGTTAAGATCACACCTAT-3'
PC	sense	5'-GATGACCTCACAGCCAAGCA-3'
	antisense	5'-GGGTACCTCTGTGTCCAAAGGA-3'
PFKB1	sense	5'-GCCTTCTAGCATACTTCCTGG-3'
	antisense	5'-TTCACAGCCTCCACATTCAG-3'
Fgf21	sense	5'-CTGCTGGGGGTCTACCAAG-3'
	antisense	5'-CTGCGCCTACCACTGTTCC-3'
Acyl	sense	5'-CAGCCAAGGCAATTTCAGAGC-3'
	antisense	5'-CTCGACGTTTGATTAACTGGTCT-3'
FAS	sense	5'-GGAGGTGGTGATAGCCGGTAT-3'
	antisense	5'-TGGGTAATCCATAGAGCCCAG-3'
GPAT4	sense	5'-AGCTTGATTGTCAACCTCCTG-3'
	antisense	5'-CCGTTGGTGTAGGGCTTGT-3'
Elovl3	sense	5'-TTCTCACGCGGGTTAAAAATGG-3'
	antisense	5'-GAGCAACAGATAGACGACCAC-3'
GPD1	sense	5'-ATGGCTGGCAAGAAAGTCTG-3'
	antisense	5'-CGTGCTGAGTGTTGATGATCT-3'
LPL	sense	5'-GGGAGTTTGGCTCCAGAGTTT-3'
	antisense	5'-TGTGTCTTCAGGGGTCCTTAG-3'
ATGL	sense	5'-GAGACCAAGTGGAACATC-3'
	antisense	5'-GTAGATGTGAGTGGCGTT-3'
PKL	sense	5'-CTTGCTCTACCGTGAGCCTC-3'

	antisense	5'-ACCACAATCACCAGATCACC-3'
PFKM	sense	5'-TGTGGTCCGAGTTGGTATCTT-3'
	antisense	5'-GCACTTCCAATCACTGTGCC-3'
GAPDH	sense	5'-TGACCACAGTCCATGCCATC-3'
	antisense	5'-GACGGACACATTGGGGGTAG-3'
GK	sense	5'-TGAGCCGGATGCAGAAGGA-3'
	antisense	5'-CTCCCAGGTCTAAGGAGAGAAA-3'
ENO1	sense	5'-TGCGTCCACTGGCATCTAC-3'
	antisense	5'-CAGAGCAGGCGCAATAGTTTTA-3'
PFKL	sense	5'-GTGGGGCAGCTTGCTGGAGG-3'
	antisense	5'-GCGGTGCAGGGCTGAGTCTG-3'
PGAM1	sense	5'-GAAAAGGGTCTTGATTGCCG-3'
	antisense	5'-GTTCATAGACGATAGGGATGCC-3'
PGK1	sense	5'-AACCTCCGCTTTCATGTAGAG-3'
	antisense	5'-GACATCTCCTAGTTTGGACAGTG-3'
TPI1	sense	5'-AACTGGAAGATGAACGGGAG-3'
	antisense	5'-GCAATTTTGGGATCCAGCTTC-3'
aP2	sense	5'-GTGATGCCTTTGTGGGAACCT-3'
	antisense	5'-ACTCTTGTGGAAGTCGCCT-3'
CideC	sense	5'-ATGGACTACGCCATGAAGTCT-3'
	antisense	5'-CGGTGCTAACACGACAGGG-3'
CD36	sense	5'-TGGCTAAATGAGACTGGGACC-3'
	antisense	5'-ACATCACCACTCCAATCCCAAG-3'
ATPCL	sense	5'-CAGCCAAGGCAATTTCAGAGC-3'
	antisense	5'-CTCGACGTTTGATTAACTGGTCT-3'
ACC	sense	5'-GAATCTCACGCGCCTACTATG-3'
	antisense	5'-ACGGTGAAATCTCTGTGCAGG-3'
SCD1	sense	5'-CGGAGACCCCTTAGATCGA-3'
	antisense	5'-TAGCCTGTAAAAGATTTCTGCAAACC-3'
HSL	sense	5'-GCTGGGCTGTCAAGCACTGT-3'
	antisense	5'-GTAACTGGGTAGGCTGCCAT-3'
UCP2	sense	5'-ACTTTCCCTCTGGATACCGC-3'
	antisense	5'-ACGGAGGCAAAGCTCATCTG-3'
FBPase	Sense	5'-AGCCTTCTGAGAAGGATGCTC-3'
	antisense	5'-GTCCAGCATGAAGCAGTTGAC-3'
Pinin	sense	5'-ACCTGGAAGGGGCAGTCAGTA-3'
	antisense	5'-ATCATCGTCTTCTGGGTCGCT-3'
Cyclophilin	sense	5'- CAGGTCCTGGCATCTTGTCC-3'
	antisense	5'- TTGCTGGTCTTGCCATTCCT-3'
EF	sense	5′-GCTAGGCCCTCTTAGACGGTTTCAGAC-3′
ER	antisense	5′-AGCTGTGTGCTTCTGTAGCAGCAACTG-3
LF	sense	5′-GACCGAGGCATACGGTACTTTTACG-3
LR	antisense	5′-ACGTCATGTCATTCTTTCCAGCCGC-3′.
Cre recombinase	sense	5'-TTGGCCCCTTACCATAACTG-3'
	antisense	5'-GAAGCAGAAGCTTAGGAAGATGG-3'
